# Grouping of chemicals into mode of action classes by automated effect pattern analysis using the zebrafish embryo toxicity test

**DOI:** 10.1007/s00204-022-03253-x

**Published:** 2022-03-07

**Authors:** E. Teixidó, T. R. Kieβling, N. Klüver, S. Scholz

**Affiliations:** 1 Department of Bioanalytical Ecotoxicology, Helmholtz Centre for Environmental Research—UFZ, Permoserstraβe 15, 04318 Leipzig, Germany; 2 GRET-Toxicology Unit, Department of Pharmacology, Toxicology and Therapeutic Chemistry, Faculty of Pharmacy and Food Sciences, University of Barcelona, 08028 Barcelona, Spain; 3 Scientific Software Solutions, 04275 Leipzig, Germany

**Keywords:** Phenotypic patterns, Classification, Sensitivity ratio, Zebrafish

## Abstract

A central element of high throughput screens for chemical effect assessment using zebrafish is the assessment and quantification of phenotypic changes. By application of an automated and more unbiased analysis of these changes using image analysis, patterns of phenotypes may be associated with the mode of action (MoA) of the exposure chemical. The aim of our study was to explore to what extent compounds can be grouped according to their anticipated toxicological or pharmacological mode of action using an automated quantitative multi-endpoint zebrafish test. Chemical-response signatures for 30 endpoints, covering phenotypic and functional features, were generated for 25 chemicals assigned to 8 broad MoA classes. Unsupervised clustering of the profiling data demonstrated that chemicals were partially grouped by their main MoA. Analysis with a supervised clustering technique such as a partial least squares discriminant analysis (PLS-DA) allowed to identify markers with a strong potential to discriminate between MoAs such as mandibular arch malformation observed for compounds interfering with retinoic acid signaling. The capacity for discriminating MoAs was also benchmarked to an available battery of in vitro toxicity data obtained from ToxCast library indicating a partially similar performance. Further, we discussed to which extent the collected dataset indicated indeed differences for compounds with presumably similar MoA or whether other factors such as toxicokinetic differences could have an important impact on the determined response patterns.

**Supplementary Information:**

The online version contains supplementary material available at 10.1007/s00204-022-03253-x.

## Introduction

Zebrafish embryos represent a promising alternative to mammalian animal models for toxicity testing (Strähle et al. 2012). They share many homologous developmental processes with mammals (Granato and Nüsslein-Volhard 1996) and many fundamental pathways involved in the response to chemicals are also highly conserved (Lieschke and Currie 2007). Regulatory organizations and industry are promoting the development and application of rapid and cost-effective assays that support mode-of-action (MoA)- or adverse outcome pathway (AOP)-based chemical screening and prioritization for further hazard characterization (Hartung 2009; OECD 2016). The zebrafish embryo model represents an alternative test system which shares the small scale of in vitro and the complexity of an in vivo test system. It allows non-invasive testing for diverse endpoints, including mortality, malformations, heart rate and behaviour (embryonic movement) without the need for sample preparation. The combination of multiple phenotypes could be used as a chemical fingerprint to identify and predict mechanisms of action or to group chemicals into classes with similar biological activity.

The integration of several morphometric parameters has been recently used to analyze the effects of specific endocrine disruptors (Martínez et al. 2019) and has also been successfully applied to identify common morphometric signatures related to teratogen exposures (Jarque et al. 2020). Several phenotypic screens using zebrafish embryos have been reported, in which the effects of chemicals or small molecules were assessed (Rihel et al. 2010; Padilla et al. 2012; Truong et al. 2014; Copmans et al. 2016; Bugel et al. 2016; Zhang et al. 2017; Thomas et al. 2019). These studies have used various approaches based on the comparison of the magnitude of an effect at a given concentration, the comparison of lowest observed effect concentrations (LOECs or LEL) or a complex integration of data for a final summary assessment. The effect assessment was often based on a scoring of the effects by an observer, causing a potential bias in the assessment. The comparison of the magnitude of an effect was also applied for embryonic behaviour assessment (Rihel et al. 2010; Copmans et al. 2016), which was conducted for single or a few concentrations of a fixed concentration range. Approaches that test for only a single or few concentrations and/or within a defined narrow range of concentrations may fail to detect effects or not capture the concentration-dependency appropriately. In contrast, the use of LOECs (lowest observed effect concentrations) used in some screens considers concentration-dependency and variability (Truong et al. 2014; Bugel et al. 2016). A disadvantage of this approach is the dependency on sample size and variability, which represents a general concern for the use of LOECs or NOECs (no observed effect concentrations), if compared to modelled effect concentrations (Jager 2012). Therefore, cellular in vitro and zebrafish embryo in vivo screening approaches such as the US-EPA ToxCast screening have used modelled effect concentrations (Knudsen et al. 2011; Padilla et al. 2012; Thomas et al. 2019).

The aim of this study was to explore to what extent compounds can be grouped according to their toxicological and or pharmacological mode of action using an automated, unbiased quantitative multi-endpoint zebrafish embryo test and determination of effect concentration ratios (i.e., comparison of specific effects to mortality and predicted baseline toxicity. Baseline toxicity is the predicted minimal mortality of a compound based on its hydrophobicity and interference with cellular membrane integrity (van Wezel and Opperhuizen 1995; Klüver et al. 2019)). Effect concentrations below the predicted baseline LC_50_ indicate interaction with additional targets and suggest specific interactions. To include an estimation of the specificity of the responses the comparative analysis was related to mortality and unspecific hydrophobicity-driven baseline toxicity. Twenty-five chemicals with known developmental toxicity and different (pharmacological) MoA were selected based on previous published studies (Teixidó et al. 2018, 2019). We hypothesized that by application of standard supervised and non-supervised descriptive statistical techniques on zebrafish embryo effect patterns chemicals can be grouped according to their MoA.

## Material and methods

### Chemicals

The list of chemicals used and physicochemical properties of relevance are described in Table 1 and table S1. All selected chemicals were active as parent compounds, i.e., they do not require metabolic activation (supplement table S2). Purity of chemicals, suppliers and final solvent concentration when stock solutions were prepared in dimethyl sulfoxide (DMSO) are given in table S1. The known or putative MoAs were obtained from a literature survey (see supplementary Table S1 for details). Stock solutions of each chemical were prepared by dissolving chemicals in embryo medium according to the OECD testing guideline 236 ((OECD 2013), pH=7.4–7.5) or in 100% DMSO. Test solutions were obtained by dilution of the stock solutions in exposure medium (2 mM CaCl_2_.2H_2_O, 0.5 mM MgSO_4_.7H_2_O, 0.75 mM NaHCO_3_ and 0.07 mM KCl, pH=7.4–7.5). Final DMSO concentrations in exposure media ranged from 0.01 to 1% DMSO in case stock solutions were not prepared directly in exposure medium. The different DMSO concentrations reflected the different maximum solubilities in DMSO. However, it was tried to keep the concentration of DMSO as low as possible. In case that DMSO was used, the same concentration was applied for all concentrations of the specific chemical and corresponding controls. The pH of the highest tested concentration ranged from or was adjusted to 7.0–8.0. For compounds with a change of proportion in neutral versus discharged form in the neutral range of pH the exact range of pH measured is given in Table S1 and was used to calculate the log *D*_*lipw*_ (pH) to obtain the predicted baseline toxicity LC_50_ for zebrafish embryos (Klüver et al. 2019).

**Table 1:** List of chemicals and corresponding physicochemical properties and specific and broad mode of action classification used for the analysis

Chemical	CAS	log *D*_*lipw*_ (pH)^a^	Mechanism of action	Broad MoA classification
Fluazifop-p-butyl	79,241-46-6	4.30	Acetyl CoA Carboxylase (ACCase) inhibitor	ACCase inhibitor
Tralkoxydim	87,820-88-0	2.15	ACCase inhibitor	ACCase inhibitor
Topiramate	97,240–79-4	3.12	GABA receptor agonist	Neuroactive
Loratadine	79,794-75-5	4.31	Histamine H1 receptor antagonist	Heart rate modulator
Acetaminophen	103-90-2	1.02	Non-selective Cyclooxygenase inhibitor	COX inhibitor
Celecoxib	169,590-42-5	4.31	Cyclooxygenase 2 inhibition	COX inhibitor
Diclofenac (obtained as sodium salt)	15,307-79-6	2.65	Non-selective Cyclooxygenase inhibitor	COX inhibitor
Firocoxib	189,954-96-9	1.90	Cyclooxygenase 2 inhibition	COX inhibitor
Oxaprozin	21,256-18-8	1.66	Non-selective Cyclooxygenase inhibitor	COX inhibitor
Olanzapine	132,539-06-1	2.0	D2 and 5HT2A antagonist	Neuroactive
Methotrexate	59-05-2	-1.0	Dihydrofolate reductase inhibitor	Antimitotic
Betamethasone (obtained as dipropionate)	5593-20-4	4.58	Glucocorticoid receptor agonist	Glucocorticoid
Dexamethasone	50-02-2	1.77	Glucocorticoid receptor agonist	Glucocorticoid
Diflorasone diacetate	2557-49-5	3.19	Glucocorticoid receptor agonist	Glucocorticoid
All-trans retinoic acid	302-79-4	4.49	Retinoic acid receptor agonist	RA signaling
Diniconazole	83,657-24-3	4.65	14 alpha-demethylase inhibitor	RA signaling
Flusilazole	85,509-19-9	4.21	14 alpha-demethylase inhibitor	RA signaling
Hexaconazole	79,983-71-4	4.17	14 alpha-demethylase inhibitor	RA signaling
Triadimenol	55,219-65-3	3.21	14 alpha-demethylase inhibitor	RA signaling
Propafenone (obtained as hydrochloride)	34,183–22-7	2.60	Sodium channel blocker	Heart rate modulator
Nortriptyline (obtained as hydrochloride)	894-71-3	3.40	Selective serotonin reuptake inhibitor (SSRI)	Neuroactive
Daunorubicin (obtained as hydrochloride)	20,830-8-13	0.05	Topoisomerase II inhibitor	Antimitotic
Carbendazim	10,605-21-7	1.70	Inhibition of microtubule assembly	Tubulin interference
Fenbendazole	43,210-67-9	3.71	Inhibition of microtubule assembly	Tubulin interference
Triclabendazole	68,786-66-3	5.93	Inhibition of microtubule assembly	Tubulin interference

References to MoA classification are provided in supplementary Table S1

^a^Calculated based on (Klüver et al. 2019), see Table S1 for details in the calculation

### Zebrafish developmental toxicity assay overview

Zebrafish developmental toxicity tests were performed similarly as previously described (Teixidó et al. 2019). Adult, healthy, unexposed zebrafish were used to produce fertilized eggs. We used the UFZ-OBI strain (generation F14-15), obtained originally from a local breeder. Fish were cultured at 26 ± 1 °C at a 14:10 h light: dark cycle in a recirculating tank system similar as described by Westerfield (Westerfield 1995). Fish were cultured and used according to German and European animal protection standards and approved by the Government of Saxony, Landesdirektion Leipzig, Germany (Aktenzeichen 75–9185.64). Embryos were exposed to the test compound, control embryo medium or solvent control from 2 h post-fertilization (hpf) during 48 and 96 h, at a temperature of 28 (± 1) °C (14:10 light:dark cycle). Forty-eight-hour exposures were conducted in crystallization dishes covered with watchmaker glasses with a test volume of 16 mL and 16 embryos per dish. Ninety-six-hour exposures were conducted in rectangular 96-well microplates (Clear Polystyrene, flat bottom, Uniplate®, Whatman™, GE Healthcare, Little Chalfont, UK) covered by a lid with a test volume of 400 µL (1 embryo per well, 16 wells per concentration tested). The different protocols were used since manual dechorionation is required for 2 days post-fertilization (dpf) embryos and is difficult to conduct in 96-well plates. The different rearing conditions between 2 and 4 dpf exposures (group exposure vs individual exposure) were considered not significant because the sensitivity between earlier and later endpoints showed a good correlation (see results section for more details). Furthermore, it has been demonstrated for instance that different rearing conditions do not alter the developmental neurotoxicity of chemicals (Zellner et al. 2011). Tests were performed with at least two replicates with changing concentrations in replicates to improve description of concentration ranges that provoked effects.

For hydrophobic compounds (i.e., loratadine with log Kow > 4) also the 96-h exposure was conducted in crystallization dishes to compensate for a potential loss of exposure concentration due to absorption in embryos and to the wells of the microplate.

Stability of the exposure solutions was confirmed (supplementary table S3) by comparison of UV/VIS spectra in the range of 200–400 nm, obtained with an EPOCH microplate reader with cuvette slot (BIOTEK, Bad Friedrichshall, Germany). In case of weak stability (> 20% loss within 96 h of exposure) renewal of the exposure solutions was performed every 24 h by replacing completely the exposure solution with freshly prepared chemical solution. For those chemicals that lack spectral properties and/or in case of interference of spectral analysis with DMSO concentrations, renewal was also conducted every 24 h. For some chemicals, stability of the exposure solutions was concluded from available literature information and no renewal was considered (supplementary Table S3).

Phenotypic assessment by automated imaging was performed after assessment of lethality (at 2 dpf and 4 dpf) and after assessment of behavioural effects (at 4 dpf). Lethality was identified by coagulation, missing heartbeat and a non-detached tail. Concentration–response curves for mortality are shown in table S4.

### Image-based quantification of morphological features

#### Automated imaging of zebrafish embryos

Embryos were dechorionated (required for 2 dpf stage only) and anesthetized with a tricaine solution (150 mg/L, TRIS 26 mM, pH 7.5). This tricaine concentration has been shown not to affect the heart rate frequency within the time frame (2 h) that was used for analysis (Yozzo et al. 2013). Embryos exposed in crystallization dishes were transferred to a 96-well microplate with rectangular wells. Images of zebrafish embryos were obtained using the VAST Bioimager (Union Biometrica, Gees, Belgium) (Pardo-Martin et al. 2010) with the on-board camera (10 µm resolution) or a coupled LEICA microscope (Leica Microsystem sDM6B equipped with a Leica digital camera DFC 365FX, Wetzlar, Germany). Loading of each fish from rectangular 96-well plates was done using the LP sampler (Union Biometrica, Gees, Belgium, settings are given in supplementary Table S5). Additionally, a video of 15 s at 30 frames per second was recorded of each embryo in lateral position for later video-based determination of the heart frequency. In cases where pictures were obtained with the LEICA microscope, two autofocus image and a Z-stack of ten images, one from the anterior and another one from the posterior part, were obtained at a magnification of 50x (Objective HCC Apo L U-U-I, 5x/0.5 water). The Z-stack was required to obtain an improved focus for the posterior and anterior part of the fish embryo using the Image J plugin “Extended depth of field” (Forster et al. 2004). Posterior and anterior images were automatically stitched together using a MATLAB script (developed by Scientific Software Solutions, www.tks3.de) embedded into a KNIME workflow (Teixidó et al. 2021). Image resolution was reduced to 30% before performing the analysis with the FishInspector software.

#### Quantification of phenotypic features

The detection and quantification of the phenotypic features after image acquisition was performed using the FishInspector software (Kießling et al. 2018) and a customized KNIME workflows as previously described in Teixidó et al. (Teixidó et al. 2019). Briefly, morphological features were annotated with the FishInspector software and the obtained JSON data files were used as input in a customized KNIME workflow with R scripts (R Core Team, 2014) to quantify the morphological features (Table 2). Shape information (mainly length and surface area) was extracted using the “Momocs'' R package (Claude et al. 2008; Bonhomme et al. 2013). In addition to the features described in Teixidó et al. (Teixidó et al. 2019), two new features were introduced, head-trunk angle and mandibular arch thickness measured at 2 dpf and 4 dpf, respectively. Head-trunk angle was measured by drawing a line between the centers of ear and eye and a second line parallel to the notochord in the mid-trunk region (Teixidó et al. 2013) using the coordinates provided by the FishInspector software. Mandibular arch thickness was calculated by measuring the distance between the eye and contour in the lower jaw using the lowest Y contour value of the eye as reference.

**Table 2:** Morphological and functional endpoints evaluated at the indicated embryo stage (days post-fertilization—dpf)

Phenotypic feature	Stage (dpf)	Parameter or metric used
Eye size	2 and 4	Surface area (mm2)
Body length	2 and 4	Distance (mm)
Yolk sac size	2 and 4	Surface area (mm2)
Otolith-eye distance	4	Distance (mm)
Head-trunk angle	2	Angle (degrees)
Pericard size	2 and 4	Surface area (mm2)
Tail curvature	2 and 4	Curvature
Swim bladder inflation	4	Surface area (mm2) and presence or absence
Head size	2 and 4	Surface area (mm2)
Pigmentation	2 and 4	Surface area (mm2)
Otoliths	2 and 4	Presence or absence (absence also includes only one otolith present)
Lower jaw position	4	Distance (mm)
Mandibular arch thickness	4	Distance (mm)
Heart rate	2 and 4	Beats per minute
Spontaneous tail coilings	1	Tail coilings/min/embryo
Locomotor response (Dark–light)	4	Mean distance travelled (Dark and light)

### Heart rate quantification

Video frames obtained with the VAST Bioimager system were analyzed using an automated image workflow developed in KNIME Analytics Platform as described by Teixidó and colleagues (Teixidó et al. 2019). The heart frequency was determined using a Fast Fourier transform of the pixel variance of the zebrafish heart region.

### Spontaneous movements

Spontaneous tail coilings (STCs) were evaluated in embryos at the age 24–25 hpf, representing the stage with the highest frequency of STCs. Coils were detected from video recordings with a duration of 1 min from exposed embryos in crystallization dishes, obtained with a camera (Olympus DP21, Shinjuku, Tokyo, Japan) mounted in a stereomicroscope (Olympus SZX7, 0.8 × magnification). Mobility of each embryo was evaluated by means of a KNIME workflow and detection was done based on variance of gray values of the individual detected embryo over time (Ogungbemi et al. 2020). The threshold for the detection of tail coiling was set by verifying concordance between the KNIME output and visual assessment of the number of STCs. Frequency of movements per min and embryo normalized to the control movement was used to calculate the effect concentrations (raw data are provided in table S6).

### Locomotor response (LMR)

The locomotor response was assessed at 4 dpf prior to the analysis of morphological phenotypes. Embryonic movement was tracked using the ZebraBox video tracking system (Viewpoint, Lyon, France) for 40 minutes in a series of light and dark periods to stimulate movement (10-min equilibration in light, followed by 20 min in dark and a final 10-min light phase) as described in Teixidó et al. (Teixidó et al. 2019). The temperature was maintained at 28 (±1) °C. We considered all live embryos, including malformed embryos and embryos showing no inflation of the swim bladder, for the analysis of the locomotor response, albeit it cannot be excluded that behaviour could be changed indirectly due to malformations. This was done since (1) not all type of malformations may impact on behaviour, (2) also malformation could change secondarily as a result of increased neuronal activity (as e.g. reported for myopathy through AChE inhibition; (Klüver et al. 2011) and (3) the number of embryos per replicate and concentration for behaviour assessment would be reduced and increase the variability. To assess specificity, we compared the effect concentrations of malformations and behaviour. Analysis of LMR data was based on the calculation of mean traveled distance in the first dark phase interval (minute 20-30) and the second light phase interval (minute 30-40), normalized to the moved distance of the control or to the moved distance of the control in the dark phase, respectively.

### Data evaluation

#### Concentration–response curves and effect concentrations

Concentration-response curves were derived for all the morphological, behavioural features and lethality as described in Teixidó et al. (Teixidó et al. 2019). The experiments were designed for calculation of effect concentrations based on curve fitting. Therefore, different concentrations were used in subsequent replicates and statistical assessment was primary aiming at securing that a concentration-dependent trend was observed. The Tukey trend test (R package “tukeytrend” (Schaarschmidt et al. 2021)) was applied to demonstrate a trend in the data obtained from at least two replicates. We also used the Akaike information criterion (AIC) to compare modeled data versus the assumption that there is no trend in the data (indicated by a lower AIC for a linear model of slope 0). Furthermore, we considered calculation of effect concentrations for a specific endpoint only if at least 30% of embryos were affected for this endpoint in at least one exposure concentration. We only used effect concentrations from modelled data sets where the indicators were appropriate (i.e., significant trend, lower AIC for the modelled response, and at least 30% of embryos affected).

Two approaches were used to determine effect concentrations based on modelled concentration-response curves: (a) use of data normalized to the mean control value and, (b) transformation to quantal effect data using a threshold. The first approach was used for endpoints with high variability between controls of replicates, observed for heart rate, behaviour and pigmentation. For these endpoints, data were normalized to the mean control of each replicate and concentration-response curves were fitted using five models: polynomial, linear, gauss, exponential and log-logistic (equations are provided in supplementary Table S7) using R with the R package drc (Ritz et al. 2015). The model for calculation of effect concentrations was selected based on the lowest Akaike information criterion (AIC). The EC_20_ (i.e., concentration with a 20% effect) was used for heart rate, pigmentation and STCs effects. For all other endpoints EC_50_ were calculated after dichotomizing the data. I.e., data were transformed to percent values by applying a threshold value established by analysis of the control variability. Histograms and the Shapiro-Wilk-Test applied to about 50 different control data sets indicated a normal distribution of features (supplement Table S8). Hence, values deviating by more than 2.5 fold of the standard deviation from controls were considered as indicating a deviation from the control and were used to calculate the fraction of embryos for which the appropriate endpoint was affected. Effect concentrations (EC_50_) were calculated based on a log-logistic model (LL.4 model from R package drc (Ritz et al. 2015)). See supplementary table S9 for information on models and effect concentrations used. In a few cases (betamethasone and diflorasone diacetate) already the lowest concentration showed high effects (more than 70% from control) on some endpoints (bladder and pericard size, respectively) and effect concentrations (EC_50_ or EC_20_) could not be calculated. In these cases, the lowest concentration was used. Supplementary table S10 contains the effect concentrations for each endpoint and chemical as well as the model used and parameters obtained.

#### Calculation of relative sensitivity ratios

The specificity and selectivity of morphological and functional endpoints was assessed by comparing the effect concentrations values (EC_50_; EC_20_ for heart rate, pigmentation and STCs) with the baseline toxicity and the lethal concentration (LC_50_) at a specific time point (2 dpf and 4 dpf). The specificity ratio (SR_baseline_) indicates how close the effect is to non-specific baseline concentration and is calculated by dividing the predicted fish embryo baseline toxicity (also referred as narcosis or minimal toxicity any chemical causes) with the EC_50_ of the sublethal effect (Eq. 1). The selectivity (SR_lethality_) is described by comparing sublethal endpoints with the experimental lethality of the corresponding stage and exposure duration (Eq. 2) (Bittner et al. 2019) and is similar to the teratogenic index used in previous studies (Selderslaghs et al. 2009). The combination of both approaches enables us to discriminate from non-specific effects and the relation of effects to potential secondary responses from overt toxicity.

1 $${SR}_{Baseline}= \frac{{LC}_{50} (baseline toxicity QSAR)}{{EC}_{50 }or EC_{20 } (experimental)}$$

2 $${SR}_{Lethality}= \frac{{LC}_{50}}{{ {EC}_{50} or EC}_{20}}$$

A value close to 1 indicates the occurrence of effects close to baseline toxicity or lethality, respectively, indicating potential non-specific responses related to overt toxicity (close to embryo death, the cells activate many cellular signaling pathways hence the exposure to high concentrations of chemicals may lead to a non-specific responses). In case no experimental LC_50_ could be obtained, the baseline toxicity of the chemical was used as a reference. For the calculation of the selectivity ratio of the spontaneous movements evaluated at 25 hpf, the LC_50_ at 2 dpf was used.

Mode of action information was extracted from literature and publicly available databases such as Drugbank (https://www.drugbank.ca/) and Hazardous Substances Data Bank (HSDB, https://pubchem.ncbi.nlm.nih.gov/). See supplementary table S1 for details.

#### Unsupervised and supervised descriptive statistics

Cluster analysis was applied using effect ratios (i.e., SR_Baseline_ or SR_Lethality_) in this study as an unsupervised statistical technique to identify clusters of chemicals on similarity of their phenotype. Effect data from 2 and 4 dpf assessment were combined to increase the number of variables and to consider endpoints that could be measured only at one of the time points. Since the data range covered multiple magnitudes, the hierarchical clustering analysis was performed using the chord distance as the similarity metric among features. This decision was made to avoid that the largest-scaled feature would dominate the others. In case no effects were observed for an endpoint, a value 0 was assigned. Ward’s method was used as a clustering method and implemented in customized R scripts using the Vegan package (Oksanen et al. 2019).

To maximize the separation between the groups and identify endpoints that allow for discrimination of groups of presumably similar acting chemicals, partial least squares-discriminant analysis (PLS-DA) was performed as a supervised statistical method. The PLS-DA is the regression extension of principal component analysis, which gives the maximum covariance between the measured data (here: effect ratio) and the response variable (group of presumably similar acting chemicals). To identify endpoints that have a strong capacity to discriminate between the different chemical classes (MoAs), variable importance in projection analysis (ViP) was performed. The PLS-DA and ViP analysis was conducted in R using the MixOmics package (Lê Cao et al. 2016).

#### Comparative analysis using in vitro test assays

In vitro biological effect data were accessed from EPA’s Comptox Chemicals dashboard (https://comptox.epa.gov/dashboard, (Williams et al. 2017)). The database contains data for ~ 10,000 compounds of hundreds of assays that were generated by the ToxCast and Tox21 in vitro high-throughput screening programs (Richard et al. 2016). Data were extracted for our set of chemicals and included both qualitative activity information (i.e., active / inactive) as well as potency estimates (i.e., AC_50_ values). However, not all chemicals were tested in all the assays, therefore in vitro assays that have not been conducted for more than 50% of the chemicals were excluded from the analysis. From the dataset the “background measurement” (measurement of each assay background noise) and also the zebrafish data were removed. The chemical concentration at half maximum efficacy AC_50_ (in μM) was used to describe effects of specific endpoints. To identify assays that show activity due to the disruption of a specific biomolecular function and not as a secondary response related to cytotoxicity we calculated the ratios of effect concentrations to cytotoxity, as an indicator of specificity of the response (SR_cytotoxicity_, Eq. 3). The SR_cytotoxicty_ was calculated similar as described by Escher et al. (Escher et al. 2020) with the cytotoxicity limit extracted from the CompTox database used as a reference value. The cytotoxicity limit is calculated as the median absolute deviation multiplied by 3 of the cytotoxicity assays as reported for the ToxCast in the CompTox database (Judson et al. 2016).

3 $${\text{SR}}_{{{\text{cytotoxicity}}}} = \frac{{{\text{Cytototxicity}}\,{\text{limit}}}}{AC_{50}}$$

For chemicals showing no activity (inactive) for a specific assay, the SR_Cytotoxicity_ was set to zero. Chemical assays used for cytotoxicity measurement were not further included in the analysis. Firocoxib was not included, because no activity data were found in the ToxCast database. Assays with no effect for any of the selected chemicals or with no data for any chemical were removed from the dataset. Multivariate analysis (PLS-DA) was performed using the same procedure used for the zebrafish phenotypic data (see section “Unsupervised and supervised descriptive statistics”).

## Results

### Developmental toxicity profile of the 25 chemicals

Zebrafish were exposed to a total of 25 chemicals in different ranges of concentrations from no effect to 100% mortality. Supplementary table S1 contains the LC_50_ values for all chemicals after 48 h and 96 h of exposure. Three compounds (dexamethasone, betamethasone and diflorasone diacetate) did not cause any mortality up to water solubility limits.

**Fig. 1:**
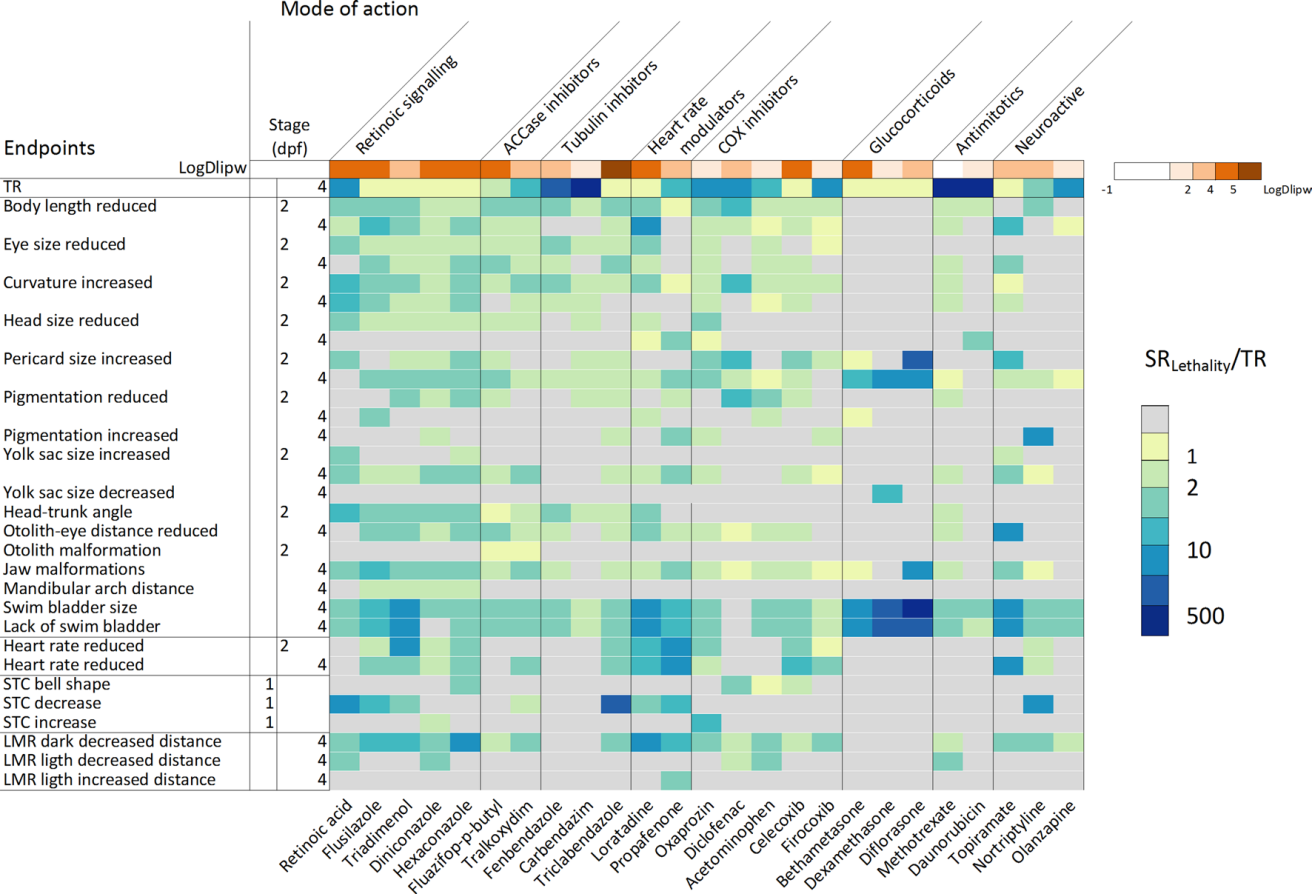
Developmental phenotypic toxicity profile of the 25 chemicals across various endpoints quantitatively evaluated using image and video analysis at 1, 2 and 4 dpf. Heatmap shows the sensitivity ratio (SR_Lethality_) from no effect (0, gray) to specific effects (500 or more, blue). The first row of the heatmap depicts the log *D*_*lipw*_ (pH) of each substance. Compounds shown in white refer to more polar chemicals with increasing intensity to orange/brown for more hydrophobic chemicals

We calculated EC_50_ values for each morphological (based on automated image analysis) and functional endpoints. For heart rate, pigmentation and spontaneous movements EC_20_ values were calculated given that these endpoints typically did not approach a 50 % effect level before mortality was observed (Supplementary Table S10). For pericard size increase and lack of swim bladder for diflorasone diacetate and betamethasone exposure, respectively, already strong effects were observed in the lowest tested concentration (>70 % of embryos were affected). Therefore, the EC_50_ was not obtained from a fitted concentration response-curve but set to the lowest tested concentration for the calculation of the sensitivity ratios. To derive effect patterns to group chemicals by their mode of action, sensitivity ratios (as SR_Lethality_) were calculated and presented as a heatmap (Fig. 1 and supplementary Table S11). Chemicals were grouped according to 8 anticipated broad mode of action groups for comparative analysis. The heatmap in Fig. 1 depicts the sensitivity ratios (SR_Lethality_) from 0 (gray, no effect) to 500 or more (dark blue) according to the pre-assigned MoA groups. The heatmap also visualizes the log *D*_*lipw*_ (pH) and toxic ratio (TR, calculated based on the baseline toxicity using the log *D*_*lipw*_ (pH)) of each chemical. The TR indicates whether mortality is caused by baseline toxicity (TR<10) or a reactive/specific mode of action (TR≥10) (Verhaar et al. 1992). TRs above ten were obtained for nine chemicals belonging to different MoA groups, mainly chemicals interfering with cell division, such as two tubulin inhibitors (fenbendazole and carbendazim), two antimitotics (daunorubicin and methotrexate), and three COX inhibitors (diclofenac, oxaprozin and firocoxib).

In general, a diversity of phenotypic responses across all the chemicals tested was observed. Failure to inflate the swim bladder or reduced swim bladder size at 4 dpf represented the most prominent effect among all substances. Due to the strong correlation between reduced swim bladder size and lack of swim bladder (see Fig. S1), we only kept the endpoint of reduced swim bladder size.

Our analysis also included functional effects like heart rate, locomotor activity and spontaneous movements. Chemicals known to have an impact on heart rate (loratadine and propafenone) provoked a specific effect on heart rate reduction with a SR_Lethality_ between 5.8 and 16.9. However, chemicals also from other MoA groups exhibited prominent effects on heart rate such as triadimenol (after 48 hours post-exposure) and topiramate (after 96 hours post-exposure) with a SR_Lethality_ of 14.7 and 33.2, respectively. Behaviour was measured by means of spontaneous movement frequencies (early in development at 25 hpf) and locomotor activity (at 4 dpf). Higher SR_Lethality_ values for reduction in locomotor activity in the dark phase were obtained for loratadine, an antihistamine and hexaconazole, a 14 alpha-demethylase inhibitor.

### Sensitivity comparison between earlier and later endpoints

**Fig. 2:**
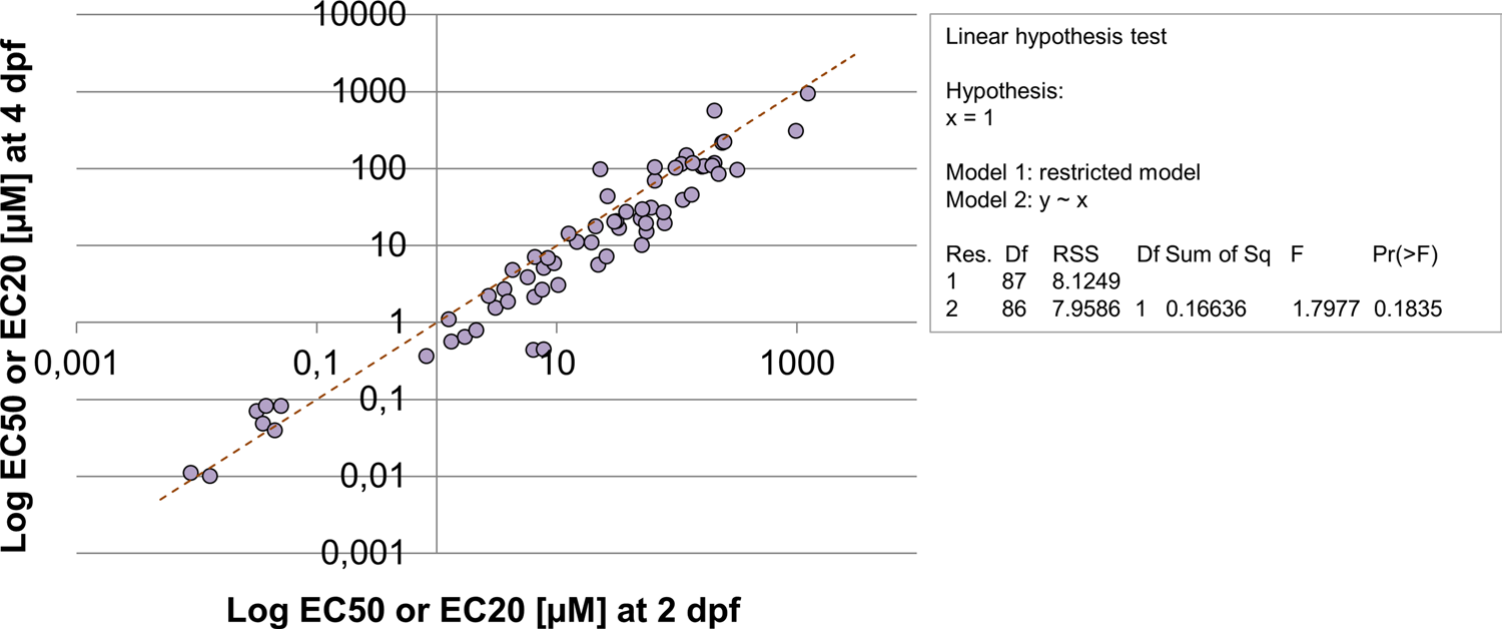
Correlation analysis of the effect concentration (EC50 or EC20-heart rate and pigmentation) between early (2 dpf, 48 h after exposure) and late endpoints (4 dpf, 96 h after exposure). The dashed line represents the line of unity

To check if early endpoints (48-h exposure) give the same information in terms of sensitivity (EC_20_ or EC_50_) as later endpoints (96-h exposure), effect concentrations only for affected endpoints in both time points were compared (Fig. 2). In general, slightly lower effect concentrations were observed after prolonged exposure time (96 h), but data showed high correlation (*r*^2^= 0.93) with a slope not statistically different from 1. However, for a few endpoints and chemicals a response was only observed for one of the time points (see supplementary Fig. S2). For instance, a reduction in head size and pigmentation were mostly detected at 2 dpf and for a few chemicals only at 4 dpf (eight chemicals vs two chemicals for head size and ten chemicals vs three chemicals for pigmentation). Exposure to Acetyl CoA Carboxylase (ACCase) inhibitors resulted in a lack otolith formation only at 2 dpf. A reduction on the yolk sac size and an increase in pigmentation were only detected at 4 dpf for 13 chemicals, and 6 chemicals, respectively (see supplementary Fig. S2).

### Specific phenotypic signatures

**Fig. 3:**
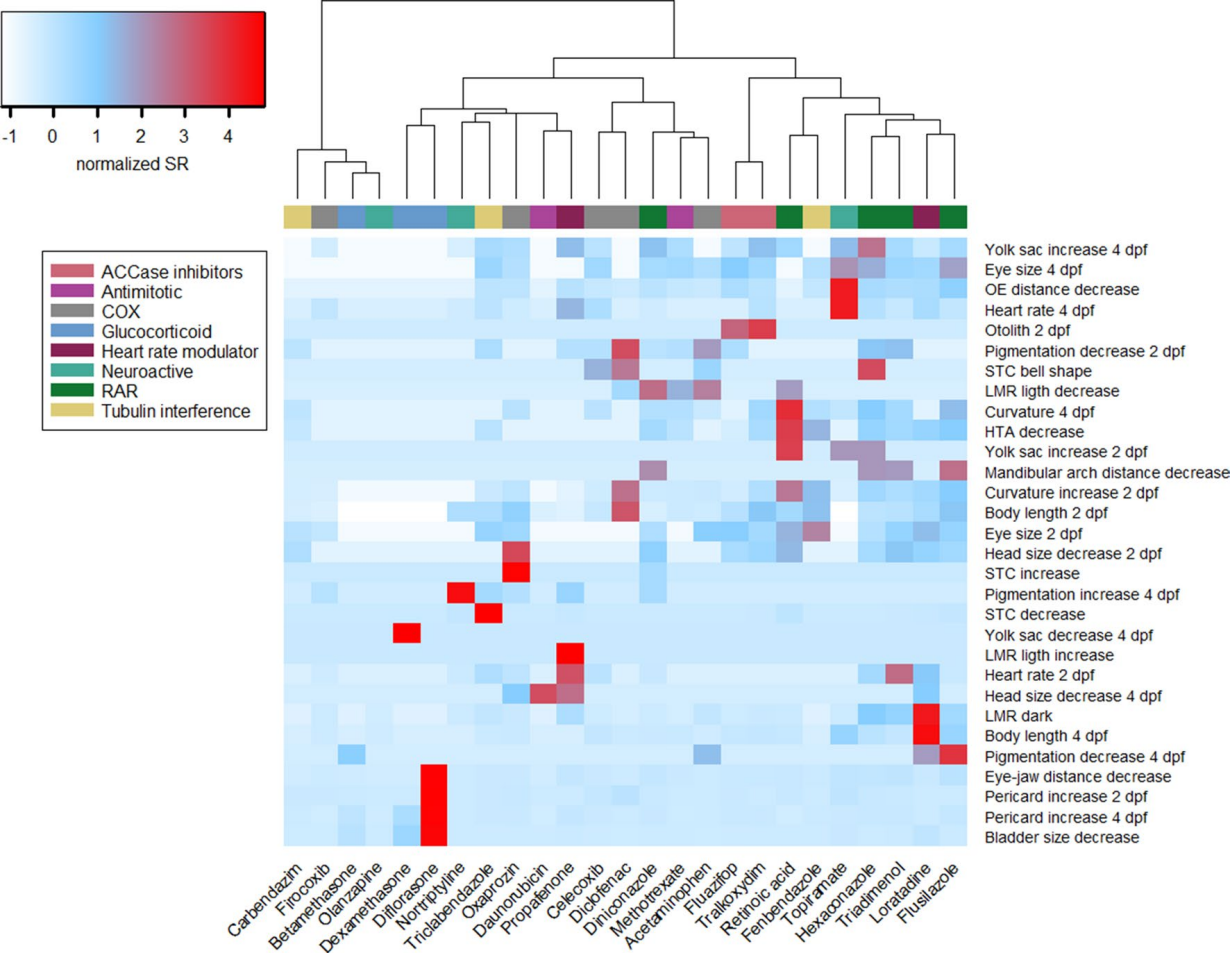
Hierarchical cluster analysis of sensitivity ratios for all endpoints. SR_Lethality_ values were scaled prior to the measurement of chord distances and clustered using Ward method. The color scale ranges from blue (low SR, normalized values) to red (high SR, normalized values). Dendrograms corresponding to the hierarchical clustering of compounds are shown on the top and supplementary Fig. S3. Clustering was performed using the gplots package in R. *RAR* chemicals interfering with the retinoic acid signalling. *COX* cyclooxygenase inhibitors

At a first glance, the visualization of sensitivity ratios (SR_Lethality_) using a heatmap appears to partially reflect the known or anticipated (primarily pharmacological) MoAs of tested chemicals (Fig. 1). However, applying hierarchical cluster analysis to the SR_Lethality_ values only grouped some of the compounds according to the assigned MoA (Fig.  3 and corresponding dendrogram in Fig. S3). Compounds that were grouped in a specific cluster were two out of three glucocorticoids (dexamethasone and diflorasone), three out of five retinoic signaling interfering compounds (flusilazole, hexaconazole and triadimenol), two out of five COX inhibitors (celecoxib and diclofenac) and the two ACCase inhibitors. Glucocorticoids displayed the most specific effects on the swim bladder. The two ACCase inhibitors showed a particular effect on otolith morphogenesis at 2 dpf (otoliths were missing or only one was present). Also three of the compounds interfering with retinoic acid signaling were grouped closely together.

Compounds belonging to the heart rate modulators, neuroactive, antimitotic and tubulin inhibitor groups were distributed among several different clusters (Figs. 3 and S3) indicating the absence of a specific phenotypic pattern for those compounds. Three of the four neuroactive compounds (nortriptyline, olanzapine, propafenone) exhibited the highest sensitivity for reduced swimming activity (supplement Tables S12 and S13). For the GABA inhibitor topiramate effects on the LMR were only shown well above the effect concentrations of many morphological features. Also some anticipated non-neuroactive compounds (all trans retinoic acid, diniconazole, firocoxib, fluazifop-p-butyl, flusilazole, hexaconazole, loratidine, tralkoxydim, triclabendazole) exhibited a high sensitivity in zebrafish embryo behaviour, particularly the locomotor response.

**Fig. 4:**
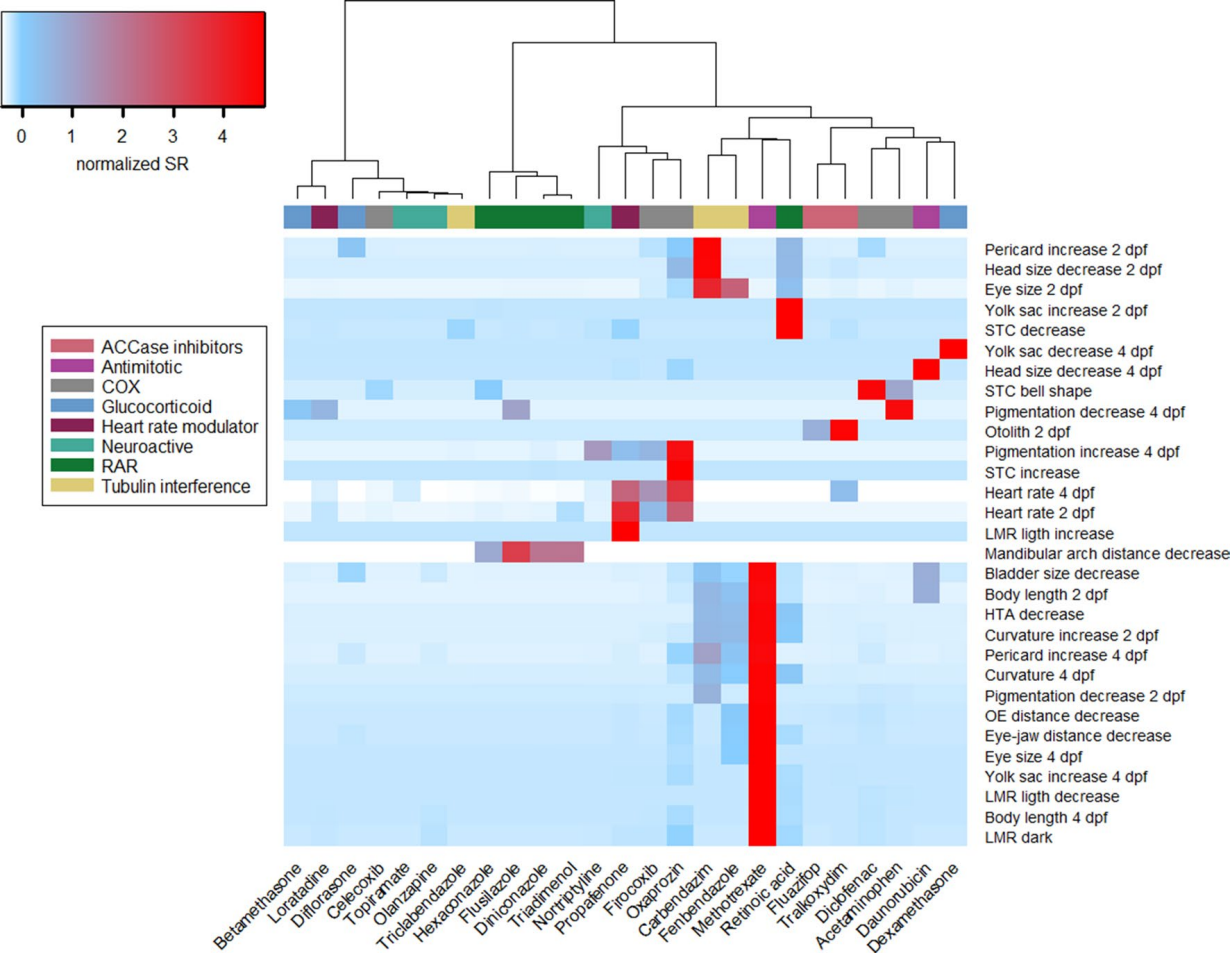
Hierarchical cluster analysis of SR_baseline_ for all endpoints. SR_baseline_ values were scaled prior to the measurement of chord distances and clustered using Ward method. The color scale ranges from blue (low SR_baseline_, normalized values) to red (high SR_baseline_, normalized values). Dendrograms corresponding to the hierarchical clustering of compounds are shown on the top and supplementary Fig. S4. Clustering was performed using the gplots package in R. *RAR* chemicals interfering with the retinoic acid signaling, *COX* cyclooxygenase inhibitors

Hierarchical cluster analysis was also performed using the sensitivity ratio calculated using the baseline toxicity of each compound (SR_Baseline_, Fig. 4 and corresponding dendrogram in Fig. S4). Results revealed that this unsupervised procedure distinguished two MoA groups: all chemicals interfering with RA signaling except the all-trans retinoic acid, and the ACCase inhibitors. Furthermore, some compounds belonging to tubulin and COX inhibitors MoA groups were grouped in specific clusters. For instance, the tubulin inhibitors carbendazim and fenbendazole exhibited high SR_baseline_ values (SR_baseline_ > 100) for several morphological endpoints like curvature, eye size and body length decrease at 2 dpf. Grouping of these compounds could also be shown by plotting the SR_Baseline_ against SR_Lethality_ for the most sensitive endpoint (see supplementary Fig. S5). For instance, compounds that interfere with cell division like the group of antimitotics or the tubulin inhibitors showed high SR_Baseline_ values (> 100) compared to the SR_Lethality_ values that were below 10. Glucocorticoids were characterized by high values of both the SR_Baseline_ and SR_Lethality_.

**Fig. 5:**
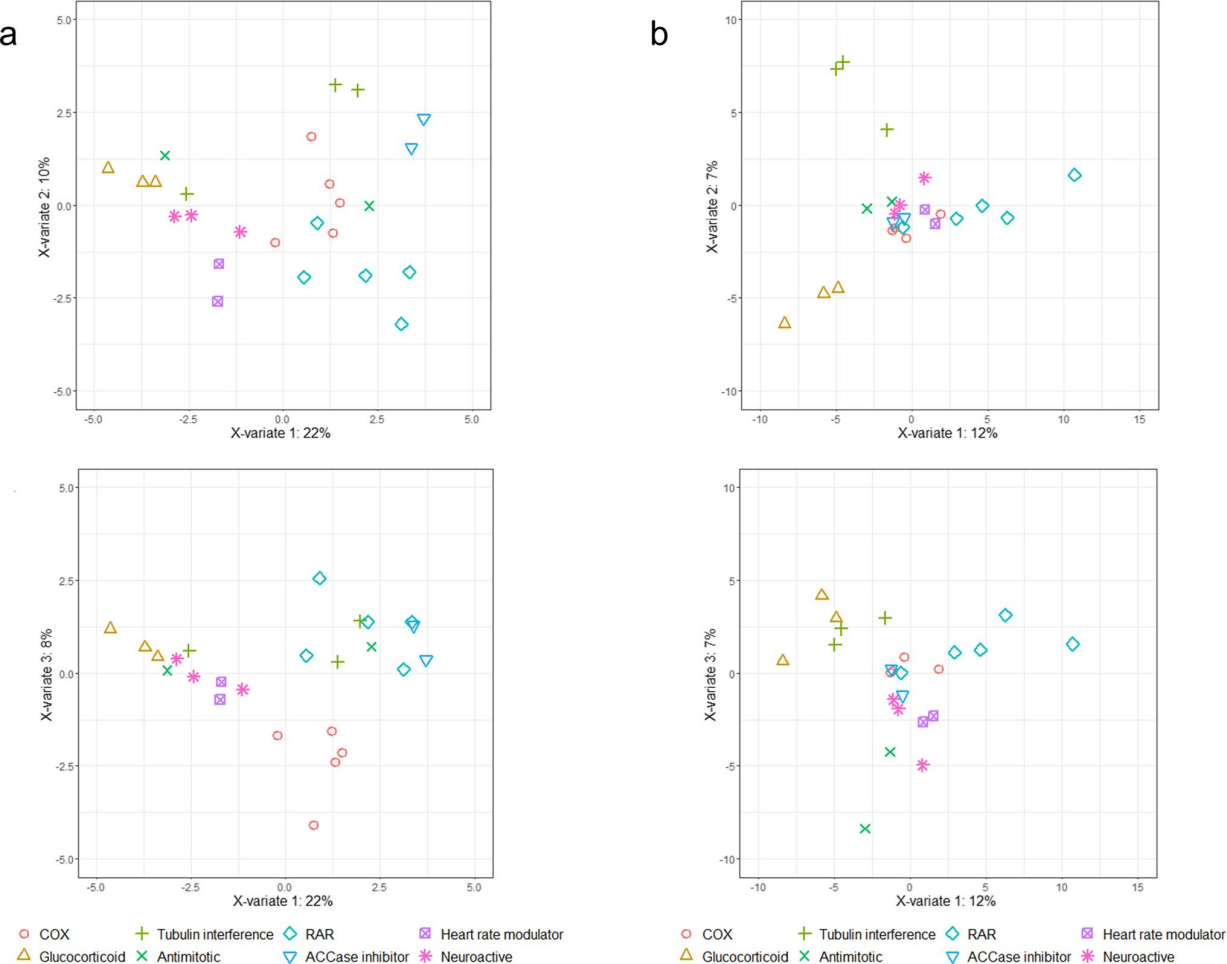
PLS-DA score plots of the tested chemicals grouped in eight broad MoA classes. Each dot represents a chemical. **A** PLS-DA score plot from SR_Lethality_ of the 30 endpoints analyzed with zebrafish between component 1 and 2 (top) and component 1 and 3 (bottom) **B** PLS-DA score plot from the SR_Cytotoxicity_ 124 in vitro assays of ToxCast library between component 1 and 2 (top) and component 1 and 3 (bottom). *RAR* chemicals interfering with the retinoic acid signaling, *COX* cyclooxygenase inhibitors

While unsupervised clustering using hierarchical cluster analysis was performed to find groups inherent to the data, supervised clustering using partial least square-discriminant analysis (PLS-DA) intended to identify endpoints with the highest discriminating power based on assigning data into eight broad MoA classes (Table 1). Fig. 5a shows the scatter plot for the PLS-DA analysis that discriminated the groups with a maximum accuracy of 50.2 % using repeated 5-fold cross-validation of 200 repeats. Differences between glucocorticoids, heart rate modulators, chemicals interfering with RA signaling and ACCase inhibitors were more pronounced than differences between other MoA groups. A similar separation but with slightly weaker accuracy (43 %) was observed for SR_Baseline_ (Supplement Fig. S6). Therefore, for subsequent analyses we focused on the use of the SR_Lethality_.

**Fig. 6:**
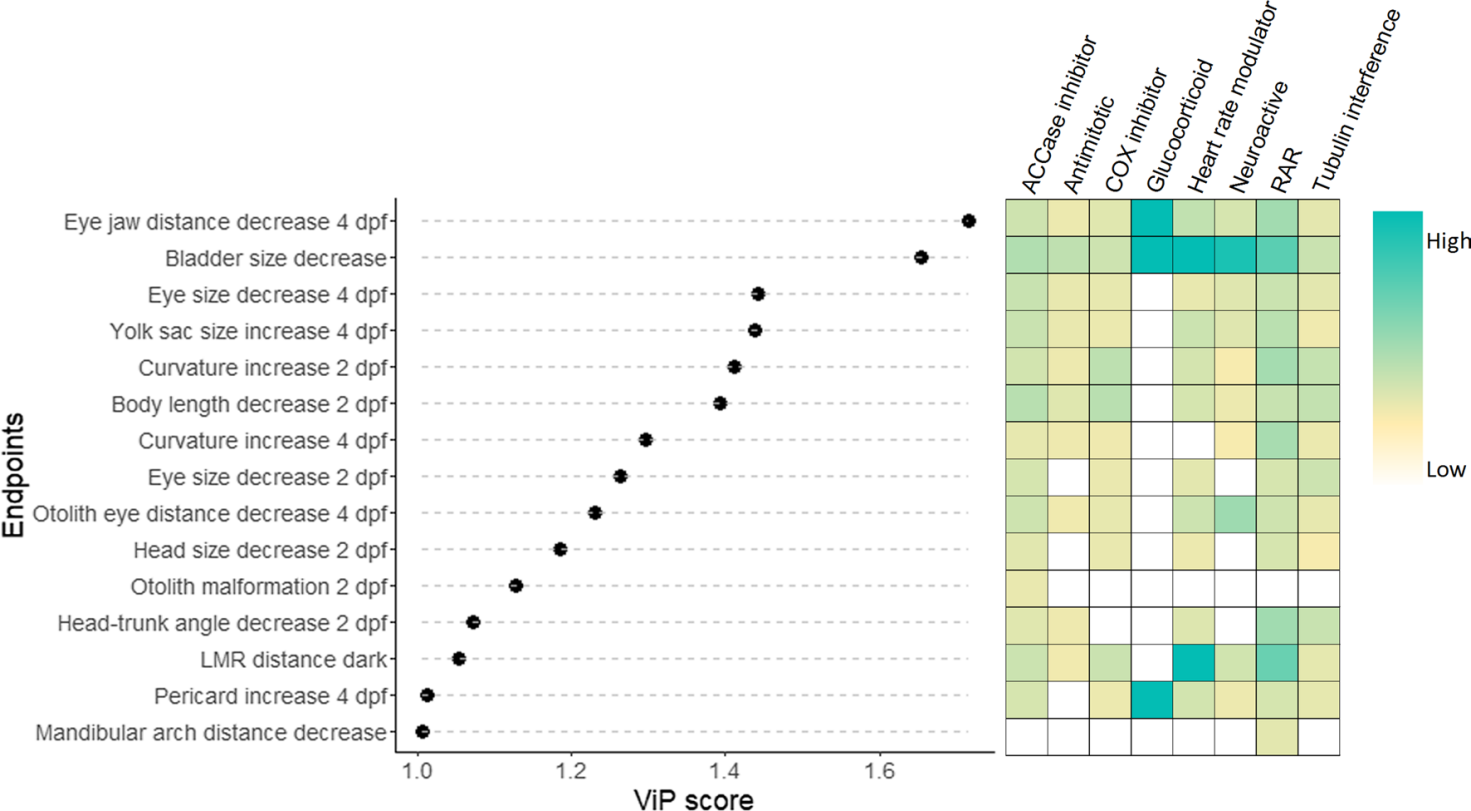
Important morphological and functional endpoints differentiating the analyzed MoA groups based on SR_Lethality_. The variable importance in projection (ViP) scores on the x-axis provide an estimate of the contribution of a given feature (shown on the y-axis) to the PLS-DA shown in Fig. 5a. The higher the ViP score, the better the morphological or functional feature is as a predictor of the discrimination among MoA groups. Colored boxes indicate the mean SR_Lethality_ of the corresponding phenotypic or functional endpoint in each MoA group. *RAR* chemicals interfering with the retinoic acid signalling. *COX* cyclooxygenase inhibitors

The identification of endpoints explaining the main differences in the MoA groups was achieved by means of using variable of importance in the projection analysis (ViP). ViP is a variable selection method, and its scores give an estimate of the contribution of a given predictor to a PLS regression model (Wold et al. 1993). ViP analysis is very useful to discard irrelevant variables, while it may have drawbacks if used for assessing the significance of features (Cocchi et al. 2018). Figure 6 shows the morphological and functional endpoints with a ViP score greater than 1, which means that a selected variable will have an above average influence on the contribution to separate the MoA group). RA interfering compounds, constituted by four triazoles and retinoic acid, represented unique group displaying a reduced mandibular arch distance. Moreover, most of the discriminatory morphological and functional endpoints displayed a high SR_Lethality_ in the group of RA interfering compounds compared to other MoA groups. Glucocorticoids showed high effects for swim bladder inflation, eye-jaw distance and pericard size increase after 96 hours of exposure (Fig. 6). The only distinctive functional endpoint among the ViP analysis was increased swimming distance in the dark period of the LMR for which the group of heart rate modulators and RA interfering compounds showed a high mean SR_Lethality_. The PLS-DA analysis was also able to identify the specific morphological effect discriminating two ACCase inhibitors from all other chemicals, i.e., the otolith morphogenesis.

### Comparison of grouping capacity with in vitro cellular assays

The discrimination of compounds in PLS-DA analysis using zebrafish embryo automated phenotype assessment was compared to the same type of analysis using published *in vitro* cellular response data from the ToxCast screening data set for two reasons. First, we wanted to indicate whether as a battery of in vitro cellular assays with specific endpoints would exhibit a similar discrimination capacity as the zebrafish embryo test assay with multiple endpoints. Second, it was intended to investigate whether the MoA groups were associated with specific biological pathways that may be involved in the developmental effects. A total of 124 assays were included in the analysis (see Table S14). Fig. 5b shows the scatter plot for the PLS-DA score using the SR_Cytotoxicity_. The analysis showed a maximum accuracy of around 31% using 5-fold cross-validation of 200 repeats attributed to a low number of chemicals analyzed for each MoA group. Despite this low accuracy, the PLS-DA plot shows that the model is able to distinguish certain MoA groups. For instance, glucocorticoids, chemicals interfering with tubulin and with the retinoic acid signaling showed a bigger separation in the PLS-DA plot figure (see Fig. 5b). Combination of our zebrafish data with the *in vitro* cellular assays gave a similar result with an accuracy of 48% (see supplementary Fig. S7).

Glucocorticoids displayed very high SR_Cytotoxicity_ for different nuclear receptor assays including the main pharmacological target, the glucocorticoid receptor (See supplementary Table S15). The group of compounds interfering with retinoic acid signaling, specially the triazoles, were the only group showing effects on aromatase inhibition (Cyp19A1, belonging to the steroidogenesis related assays) with a SR_Cytotoxicity_ of around 6. COX inhibitors displayed also high SR_Cytotoxicity_ for several nuclear receptor assays such as androgen, estrogen and peroxisome proliferator-activated receptors (PPAR), and also the transcription factor NF-κB (see supplementary Table S15). Lack of ability to discriminate between certain MoA groups using ToxCast data could, however, also result from incomplete coverage of chemicals by some of the assays. For instance, the main pharmacological target for COX-2 and COX-1 inhibitors was only analysed for 5 out of the 23 compounds subjected to ToxCast analysis (see supplementary Table S16).

## Discussion

In this study, we focused on the applicability of the zebrafish embryo test to explore how quantitative analysis of phenotypic data can be leveraged to obtain information about their MoA or similarity in the MoA of chemicals. The assessment was applied to a relatively small set of 25 chemicals as a proof-of-concept analysis and to identify potential limitations and requirements for amendments of this approach before it is applied to a large-scale grouping of chemical effects. A crucial aspect of the analysis was to obtain data from an unbiased assessment using automated image analysis (Teixidó et al. 2019) which enabled to obtain reproducible concentration-response relationships for individual phenotypes. The selected 25 test chemicals were classified in to 8 different broad MoA groups, mainly based on the known pharmacological MoA that may not necessarily be responsible for phenotypic effects. The developmental profile of these chemicals using sensitivity ratios showed that the most prominent effect among all substances was failure to inflate the swim bladder or reduced swim bladder size. The swim bladder is an air-filled organ responsible to mainly regulate the buoyancy of the fish in the water. Its development starts at 35 hpf with tissue formation and functioning is established between 96 and 120 hpf with its inflation (Winata et al. 2009). The development of swim bladder also largely depends on blood circulation (Yue et al. 2015) and could be sensitive to reduced heart rate (Bittner et al. 2018). Our study supports a potential link to blood circulation, given that swim bladder size showed a positive correlation (*R*^2^= 0.57–0.99) with heart rate for 6 chemicals out of 11 chemicals for which heart rate was affected at 4 dpf (See supplementary Fig. S8). Given that effects were assessed within 4 dpf, inflation of the swim bladder is still in progress and hence a reduced inflation may also reflect a slight delay in development. Therefore, swim bladder size also showed a significant positive correlation with endpoints related to developmental rate like otolith-eye distance (*R*^2^ = 0.67–0.92, for seven out of ten chemicals, Fig. S8). Some studies also suggested that swim bladder inflation could be delayed due to the mechanical pressure carried out by bulky yolk sac remnants (Raldúa et al. 2008). Our study also showed an inverse correlation between yolk sac area and swim bladder size (*R*^2^ between − 0.78 and − 0.95, for 7 out of 16 chemicals, Fig. S8). Particularly the MoA group of glucocorticoids showed a high SR_Lethality_ for this endpoint.

In our study, we also analysed functional endpoints such as heart rate (at 2 and 4 dpf) and behaviour (at 25 hpf and 4 dpf). Higher SR_Lethality_ values for behavioural endpoints were observed for loratadine, an antihistamine, and hexaconazole, a 14 alpha-demethylase inhibitor. Earlier studies have revealed deviating results with regards to a link of behavioural effects and histamine activity (Sundvik et al. 2011; Chen et al. 2017). Off-target effects of antihistamines by an antimuscarinic activity could influence behaviour and heart rate control (Bittner et al. 2019). However, phenotypic abnormalities could also contribute to swimming impairment (Padilla et al. 2011; Legradi et al. 2014) and in the case of loratadine, reduced swimming activity occurred when body length and swim bladder size were already compromised.

The lack in grouping of neuroactive compounds could result from the weak sensitivity of behaviour relative to morphological effects of the GABA inhibitor toparimate and high sensitivity of several anticipated non-neuroactive compounds for locomotion effects. Other GABA inhibitors such as endosulfane have been principally shown to impact on locomotion in zebrafish embryos (Klüver et al. 2015). The relative weak sensitivity of behaviour for topiramate may indicate a weak target affinity in zebrafish and/or relate to the high teratogenic potential in zebrafish described earlier (Jarque et al. 2020). Furthermore, it has been previously shown that behavioural alterations may not be restricted to neuroactive compounds (Leuthold et al. 2019). In agreement with this observation, in our study several anticipated non-neuroactive compounds showed a high sensitivity for behaviour effect. All COX inhibitors except one showed an increase in spontaneous movements with SR_Lethality_ ranging from 0.89 to 9.57. It is currently not well understood what type of non-neuroactive modes of action (excluding morphological changes) may also impact on behaviour or whether these results may indicate unknown impacts on nervous system function and development. Since our mode of action classification is based on the known (human) pharmacological mode of action, the observed behavioural effects could have been caused by off-target interactions.

The sensitivity comparison between earlier and later endpoints showed a high correlation with a slope not statistically significant different from 1. Therefore, restriction of analysis to one time point may result in a similar diagnostic value. Assessment after 96 h may be preferred over 48 h given that additional endpoints such as swim bladder inflation and locomotor response assessment are available for the later time point. Furthermore, assessment of effects at only 4 dpf offers technical advantages, given that no dechorionation of embryos is required.

Unsupervised clustering analyses provides a way to discover similarities in effect patterns. This unsupervised cluster analysis revealed that only some compounds belonging to the same assigned MoA group appeared to share similar phenotypic patterns. Using the SR_lethality_ and SR_baseline toxicity_ as indicators of specificity of the responses we were able to identify phenotypic clusters for glucocorticoids, COX inhibitors, compounds interfering with RA signaling and/or the ACCase inhibitors. Not all endpoints analyzed may be informative and related to the MoAs of the test chemicals. Therefore, a supervised assessment could indicate whether in principle the pre-assigned MoA classes could be discriminated by the effect data. However, also the supervised approach resulted only in a partial grouping of the chemicals with the same assigned MoA. Using the ViP analysis, we identified 15 endpoints that could be used for practical discrimination of phenotypes between the different MoA groups analyzed. Some of the endpoints with the strongest discriminating power were linked to known mechanisms of the test chemicals. For instance, for compounds interfering with RA signaling the lower mandibular thickness was separating this group form other compounds. Several studies suggest that triazoles might interfere with RA levels due to their inhibitory effect on the expression of CYP enzymes of the CYP26 family (Menegola et al. 2006; Marotta and Tiboni 2010). Triazoles partially inhibit CYP26A1, and likely the other CYP26 members as well, which then results in locally increased ectopic levels of RA. This especially affects the caudal region containing the posterior growth zone and the hindbrain (White et al. 1997). In addition, disturbed RA signaling in the branchial arches may contribute to the craniofacial deformities as well, as Cyp26 enzymes are expressed in the branchial arches (Rhinn and Dollé 2012).

Previous studies based on principal component analysis of chemical treated embryos and scored developmental effects indicated that much of the separation between chemicals was related to a lack of any response, with mortality as the only observed effect, or multiple endpoints affected simultaneously (Truong et al. 2014). Partially a strong correlation between endpoints was observed. In our study, this was observed for correlation of the swim bladder inflation with other endpoints such as heart rate and yolk sac size.

The partial clustering along the assigned MoA may have been caused by several reasons. First, for compounds of the same MoA, differences in the uptake and time point when internal equilibrium is approaching could result in different morphological phenotypes. I.e., effective concentrations in early windows of sensitivities may not have been reached or been masked by effects caused in later developmental stages. Since zebrafish embryos were exposed via the water, the time needed to reach steady-state internal concentration of a chemical can vary depending on the physicochemical properties, particularly their hydrophobicity (as indicated by log *D*_*lipw*_ (pH)). Strong differences in the uptake of chemicals in the zebrafish embryo model have been previously shown by assessment of internal concentration time courses (Massei et al. 2015; Brox et al. 2016). Hence, chemicals belonging to the same MoA group with different toxicokinetic properties may lead to different phenotype patterns due to internal concentration time course differences. In our study, for instance, the group of COX inhibitors displayed a very wide range of log *D*_*lipw*_ (pH) values (from 1.02 for acetaminophen to 4.31 for celecoxib, Table S1). These chemicals were distributed among several different clusters (Fig. 3). In contrast the compounds interfering with the retinoic acid signaling were clustered together, but also exhibited a narrower log *D*_*lipw*_ (pH) range (between 3.2 and 4.6) (Figs. 3 and 4). Second, our assignment of MoA groups was based mainly on the pharmacological MoA. However, the observed phenotypic effects may not have been caused by the pharmacological MoA but by (unknown) multiple compound-specific off-target MoA (or a combination of pharmacological and off-target effects) with molecular pathways leading to different developmental and functional effects. Furthermore, the pharmacological MoAs may be only relevant in a narrow concentration window and the observed phenotypic effects may relate to other off-target MoA occurring at higher concentrations and driving the phenotypic effect patterns. For instance, it is known that glucocorticoids particularly at higher concentration can provoke non-genomic effects and non-specific effects with cellular membranes (Strehl et al. 2011). For compounds interfering with retinoic acid signalling—except for retinoic acid (Zeng et al. 2017)—this is unlikely to be relevant as the tested triazoles are known to interfere with internal retinoic acid metabolism and levels (Menegola et al. 2006; Marotta and Tiboni 2010). The potential bias by grouping into pharmacological MoA was also supported by the comparative assessment of ToxCast *in vitro* data, since difference in the uptake may play a minor role for cellular models (Vogs and Altenburger 2016). For instance, the ToxCast data showed that COX inhibitors affected multiple signaling pathways (androgen, estrogen, PPAR and transcription factor NF-κB, see supplementary table S15), effects that have been linked to some COX inhibitors (Mitchell et al. 1997; Inoue et al. 2000; Kashiwagi et al. 2013). Hence, the activation of other biological pathways unrelated to their main pharmacological target, may indicate that the differences in response patterns rather reflects dissimilarities for compounds with a presumably similar mode of action. Another possibility is that it may not be possible to rely solely on phenotypes to classify even broad mode of action of chemicals in zebrafish embryo. The variability and specificity of developmental effects may preclude chemical classifications and may require considering additional endpoints, e.g., at the molecular response level.

Our study has demonstrated how the combination of supervised and unsupervised techniques from an unbiased non-invasive assessment of morphological and behavioural endpoints in an organismal alternative test system can be used to cluster compounds according to their potential mode of action. The capability of the zebrafish to recognize consistent patterns of developmental toxicity was particularly demonstrated for certain MoAs, such as the interference with retinoic acid signaling or the glucocorticoid pathway. Moreover, the comparative assessment with the in vitro ToxCast assays showed that a single test system such as the zebrafish embryo but with multiple endpoints and high-content assessment may have a similar discriminating capacity as an in vitro test battery. A potential application of the demonstrated approach is for screening of compounds and assigning a potential MoA of developmental toxicity to unknown chemicals and prioritizing further testing. Additional research is needed to assess potential factors that impact on effect patterns or to confirm whether differences in compounds with presumably similar MoA were indeed provoked by different MoAs.

## Supplementary Information

Below is the link to the electronic supplementary material.

Supplementary file1 (XLSX 1189 KB)

Supplementary file2 (PDF 501 KB)
