# Supplementary material for: Grouping of chemicals into mode of action classes by automated effect pattern analysis using the zebrafish embryo toxicity test
Source: Arch Toxicol. 2022 Mar 7;96(5):1353–69. doi: 10.1007/s00204-022-03253-x (PMC9013687; doi:10.1007/s00204-022-03253-x)
Supplement: Supplementary file 2 — Supplementary file2 (PDF 501 KB) [file 204_2022_3253_MOESM2_ESM.pdf]

SUPPLEMENTARY MATERIAL

Grouping of chemicals into mode of action classes by automated effect pattern analysis using the zebrafish embryo toxicity test

Authors: Teixidó E.[1,2], Kieβling T.R.[3], Klüver N.[1], Scholz S.[1]

[1] Department of Bioanalytical Ecotoxicology, Helmholtz Centre for Environmental Research—UFZ, Permoserstrasse 15, Leipzig 04318, Germany

[2] GRET-Toxicology Unit, Department of Pharmacology, Toxicology and Therapeutic Chemistry, Faculty of Pharmacy and Food Sciences, University of Barcelona, 08028 Barcelona, Spain

[3] Scientific Software Solutions, Leipzig 04275, Germany

Tables

Tables S1, S3-S4 and S6, S7-S16 are provided as an Excel file.

Table S1. List of chemicals and additional physicochemical properties (LogS, log K_{ow}) and calculation of log D_{lipw}(pH). Suppliers and purity. Calculated baseline toxicity and toxic ratio for chemicals.

Table S2. Parent and metabolite compound activity of the selected test compounds. All chemicals are primary toxicants and do not require metabolic activation.

Chemical	Type of chemical	Active metabolite identified	Comment	Reference
Loratadine	Drug	Descarboethoxyloratadine	4 times more potent than loratadine	(Barenholtz and McLeod 1989)
Diclofenac	Drug	4'-hydroxy diclofenac	Very weakly active (one thirtieth the activity of diclofenac)	(Davies 1998)
Propafenone	Drug	5-hydroxypropafenone and N-depropylpropafenone	These metabolites have antiarrhythmic activity comparable to propafenone but are present in concentrations less than 25% of propafenone concentrations	(Hii et al. 1991)
Nortriptyline	Drug	10-hydroxynortriptyline	1/8 the activity of nortriptyline	(Nordin and Bertilsson 1995)
Daunorubicin	Drug	Daunorubicinol	Display biological activity in addition to daunorubicin	(Takanashi and Bachur 1975)
All-trans retinoic acid	Drug	Hydroxylated metabolites and 4-oxo-atRA	Display biological activity in addition to atRA	(Topletz et al. 2015)
Triclabendazole	Drug	Triclabendazole sulfoxide	Display biological activity in addition to TCBZ	(Wishart et al. 2018)
Acetaminophen	Drug	No active metabolite		(Wishart et al. 2018)
Betamethasone	Drug	No information found		
Carbendazim	Fungicide	No active metabolite	Active metabolite of Benomyl	(JMPR, 1973)

Celecoxib	Drug	No active metabolite		(Wishart et al. 2018)
Dexamethasone	Drug	No information found		
Diflorasone	Drug	No information found		
Diniconazole	Fungicide	No information found		
Fenbendazole	Drug	No active metabolite		(Wishart et al. 2018)
Firocoxib	Drug	No active metabolite		(Kvaternick et al. 2007)
Fluazifop-p-butyl	Herbicide	No information found		
Flusilazole	Fungicide	No information found		
Hexaconazole	Fungicide	No information found		
Methotrexate	Drug	7-Hydroxymethotrexate		(Lankelma et al. 1980)
Olanzapine	Drug	N-desmethyl olanzapine and 2-hydroxymethylolanzapine	Less active than parent compound	(Calligaro et al. 1997)
Oxaprozin	Drug	No active metabolite		(Wishart et al. 2018)
Topiramate	Drug	No active metabolite		(Wishart et al. 2018)
Tralkoxydim	Herbicide	No information found		
Triadimenol	Fungicide	No information found		

References

Barenholtz HA, McLeod DC (1989) Loratadine: A nonsedating antihistamine with once-daily dosing. DICP, Annals of Pharmacotherapy 23:445–50.

Calligaro DO, Fairhurst J, Hotten TM, et al (1997) The synthesis and biological activity of some known and putative metabolites of the atypical antipsychotic agent olanzapine (LY170053). Bioorganic & Medicinal Chemistry Letters 7:25–30.

Davies NM (1998) Clinical Pharmacokinetics of Oxaprozin. Clinical Pharmacokinetics 35:425–436.

Hii JTY, Duff HJ, Burgess ED (1991) Clinical Pharmacokinetics of Propafenone. Clinical Pharmacokinetics 21:1–10.

JMPR, 1973. IPCS INCHEM. Carbendazim (WHO Pesticide Residues Series 3). https://inchem.org/documents/jmpr/jmpmono/v073pr11.htm

Kvaternick V, Pollmeier M, Fischer J, Hanson PD (2007) Pharmacokinetics and metabolism of orally administered firocoxib, a novel second generation coxib, in horses. Journal of Veterinary Pharmacology and Therapeutics 30:208–217.

Lankelma J, van der Klein E, Ramaekers F (1980) The role of 7-hydroxymethotrexate during methotrexate anti-cancer therapy. Cancer letters 9:133–142.

Nordin C, Bertilsson L (1995) Active Hydroxymetabolites of Antidepressants: Emphasis on E-10-Hydroxy-Nortriptyline. Clinical Pharmacokinetics 28:26–40.

Takanashi S, Bachur NR (1975) Daunorubicin metabolites in human urine. Journal of Pharmacology and Experimental Therapeutics 195:41–49

Topletz AR, Tripathy S, Foti RS, et al (2015) Induction of CYP26A1 by metabolites of retinoic acid: Evidence that CYP26A1 is an important enzyme in the elimination of active retinoids. Molecular Pharmacology 87:430–441.

Wishart DS, Feunang YD, Guo AC, et al (2018) DrugBank 5.0: A major update to the DrugBank database for 2018. Nucleic Acids Research.

Table S3. Chemicals and renewal regime performed accordingly to the confirmation of stability of exposure solutions

Table S4. Concentration-response curves for mortality in zebrafish embryos exposed for 48 and 96 hours. LC_{10} and LC_{50}s were obtained from modelled concentration response curves. RSS (residual sum of square) is used as an indicator of the goodness of the fit. Modelling is only conducted in case of a concentration dependent increase of mortality.

Table S5. Settings of the VAST Bioimager and LAS software (Leica Biosystems). Software version 1.2.5.1.

**Loading setup**		**Centering fish inside the capillary**	
Max volume between flushes	300 µL	Displacement with 5 pump microsteps	1 mm
Initial pluger position	1000 µL	Min. visible fish length	3.9 mm
Limit for loading volume	1000 µL	Delay before and after adjusting position	700 ms
Attempt to re-load	off	Number of attempts	2
Re-loading backup volume	40 µL		
Unloading setup (one fish at a time)	Move forward after backup	**Backup**	
Push through volume	80 µL	Max backup volume	30 µL
Unloading volume 1	30 µL	Min backup volume	1 µL
Push-back volume	40 µL	Volume of a first backup pulse	2 steps
Unloading volume 2	400 µL	Move forward after backup	head first 7 microsteps, tail first 7 microsteps
Unload multiple fish in bulk	off		
Operational mode setup	Rotational angle	**Object detection and rotation setup**	
Rotational position	Auto	Drop of intensity threshold	20
Hi-Resolution imaging	Auto	Min average intensity	130
		Min intensity drop	25%
Output		Bubbles and debris	detect and discard
Bubbles and debrie	Manual	Manual	90
Auto mixer	On	Minimum similarity	0.6
Speed	10%	Rotational backlash	5 degrees
Oscil	3 (sec)		

**Camera settings**		**Pump speeds**	
Exposure (Manual)	380 µ ├s	Loading speed	120
Gain (Manual)	1 dB	Stepping speed	2
		Backup speed	70
**White balance**		Unloading speed	2000
Red Gain	170%	Stepping speed	50
Blue Gain	200%	Flushing speed	4000
		Priming speed	2000
**Imaging with external device**		Wand aspirate speed	2000
X-position	290 µm	Backlash (microsteps)	14
Rotation angle	180		
Ctrl output	1		
Trigger output	1		
Bright field	off		
Tray LED	off		
Trigger out delay	700 ms		
Trigger timeout	30000 ms		
Degree per rotation	360		
Rotation speed	30		
CSV file record	on		

Table S6. Raw data on frequency of spontaneous tail coilings for all chemicals.

Table S7. Model equations used for modelling concentration-response curves.

Model type	Equation	Parameters
Hill (sigmoidal 4 parameter equation)	LL.4 See drc package in r[a]	
Gauss	Gaussian see drc package in r[a]	
Exponential	EXD.3 see drc package in r[a]	
Polynomial	$y = x + bx^2$	b slope
Linear	$y = d + bx$	b slope<br>d mean control value

[a]drc package documentation: https://cran.r-project.org/web/packages/drc/drc.pdf

Table S8. Histograms and Shapiro-Wilk-T test results from control data at 2 and 4 dpf.

Table S9. Magnitude of effect concentrations used according to endpoint

Table S10. Effect concentrations and concentration-response curves parameters in zebrafish embryos for all endpoints and chemicals.

Table S11. Sensitivity ratios for all chemicals across all evaluated endpoints

Table S12: Comparison of EC10 for morphological (4 dpf) and behaviour endpoints (LMR and STC)

Table S13: Comparison of EC50 for morphological (4 dpf) and behaviour endpoints (LMR and STC)

Table S14. Calculated $SR_{Cytotoxicity}$ for each assay of the Comptox library included in the analysis

Table S15. Heatmap of the mean $SR_{Cytotoxicity}$ values of each MoA group for each target subfamily ToxCast assay included

Table S16. Activity reported for our set of chemicals on the COX-1 and COX-2 activity assays in the Comptox library

Figures

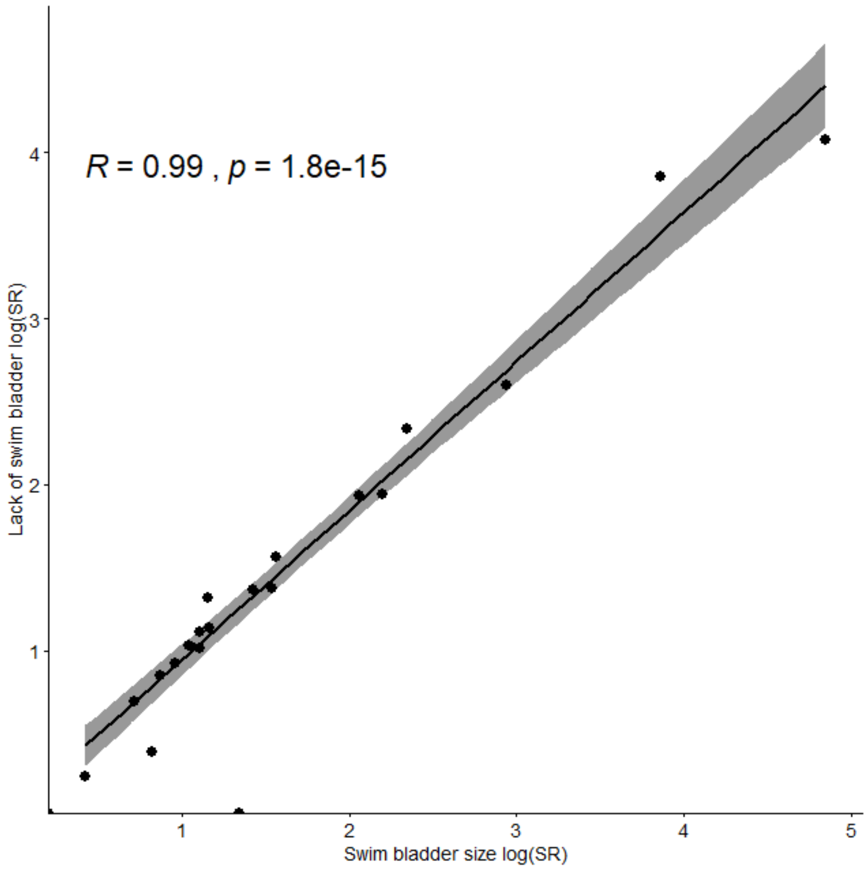

Fig. S1. Correlation between SR of swim bladder size and lack of swim bladder for all chemicals.

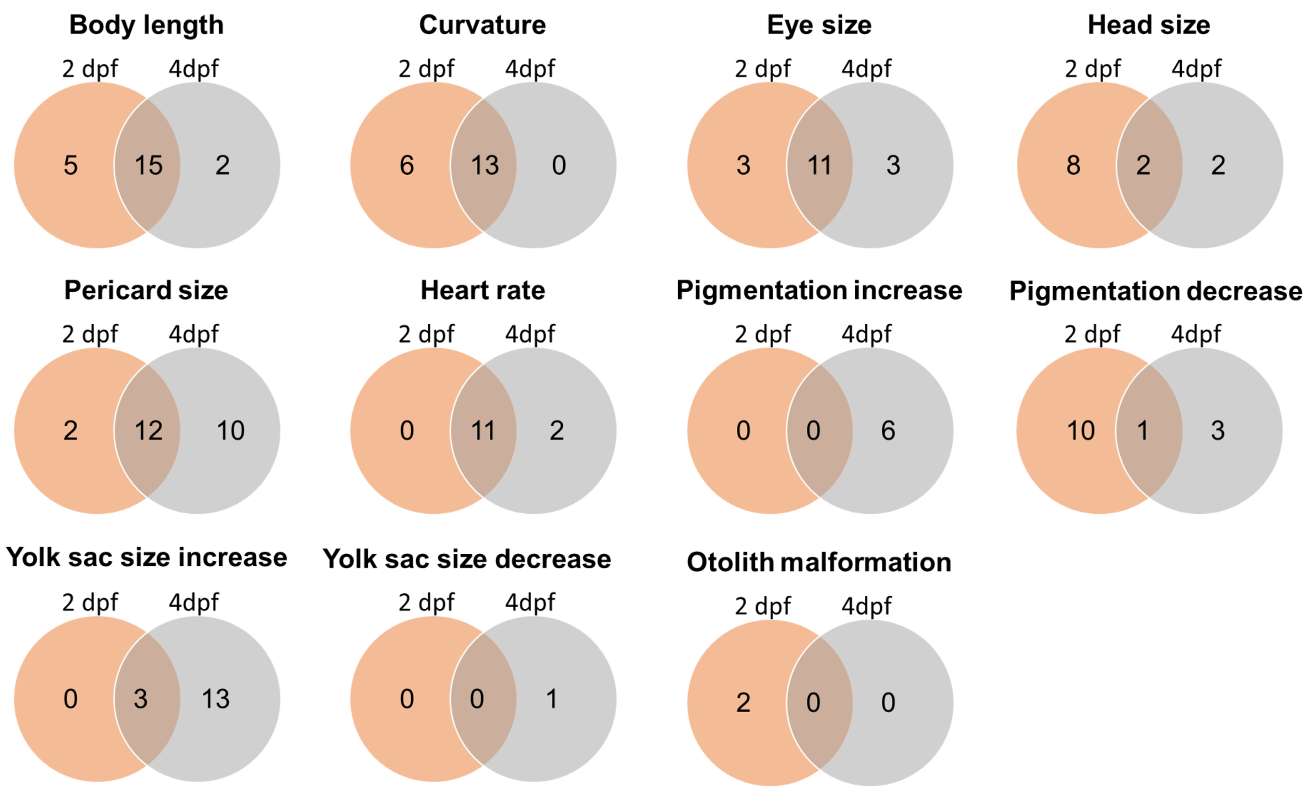

Fig. S2. Venn diagrams of chemicals showing effects at 2 dpf and 4dpf. The number of chemicals in each time point and the overlap between time points for each endpoint analysed are shown in the Venn diagrams. The only chemical showing a yolk sac size decrease at 4 dpf was dexamethasone and the two chemicals showing otolith malformations at 2 dpf were the Acetyl CoA Carboxylase (ACCase) inhibitors (tralkoxydim and fluazifop).

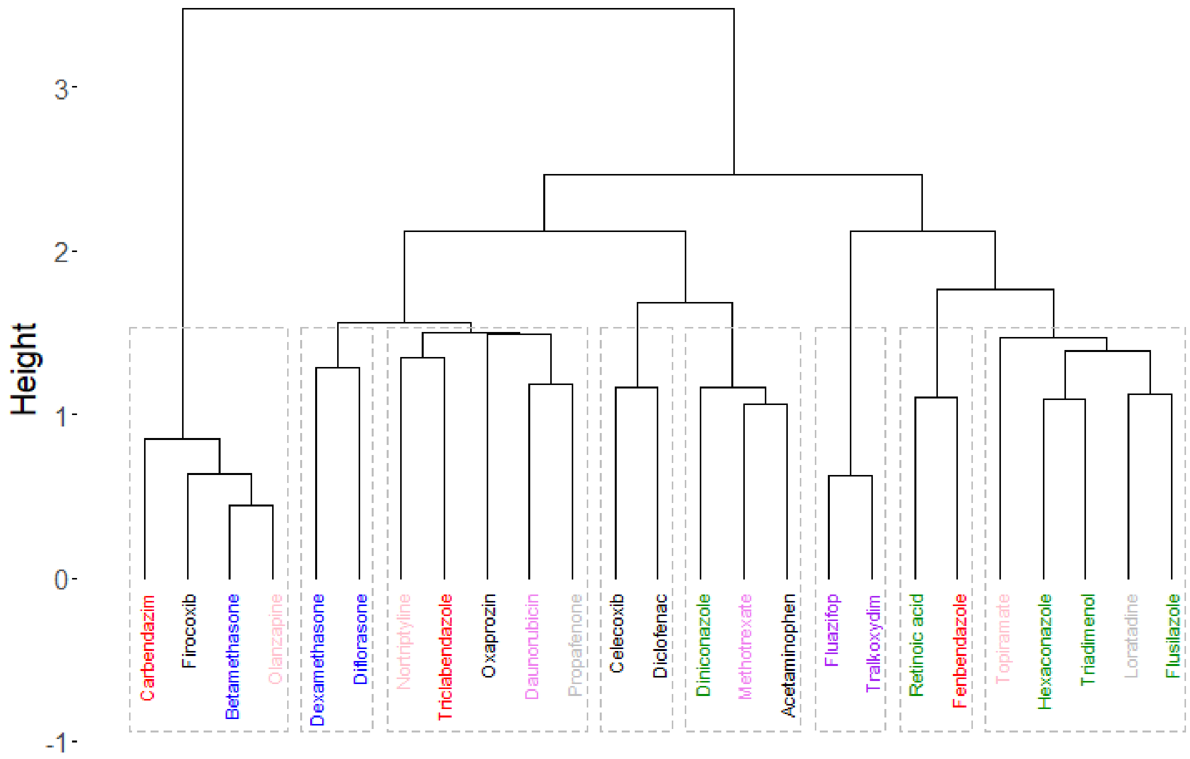

Fig. S3. Dendrogram of the cluster analysis using chemical $SR_{Lethality}$ based on the chord distance and ward method. Chemical names are colored according to their MoA group. (black: COX inhibitors, blue: glucocorticoids, red: tubulin inhibitors, purple: ACCase inhibitors, violet: antimitotics, grey: heart rate modulators, green: retinoic signaling interfering compounds, pink: neuroactive)

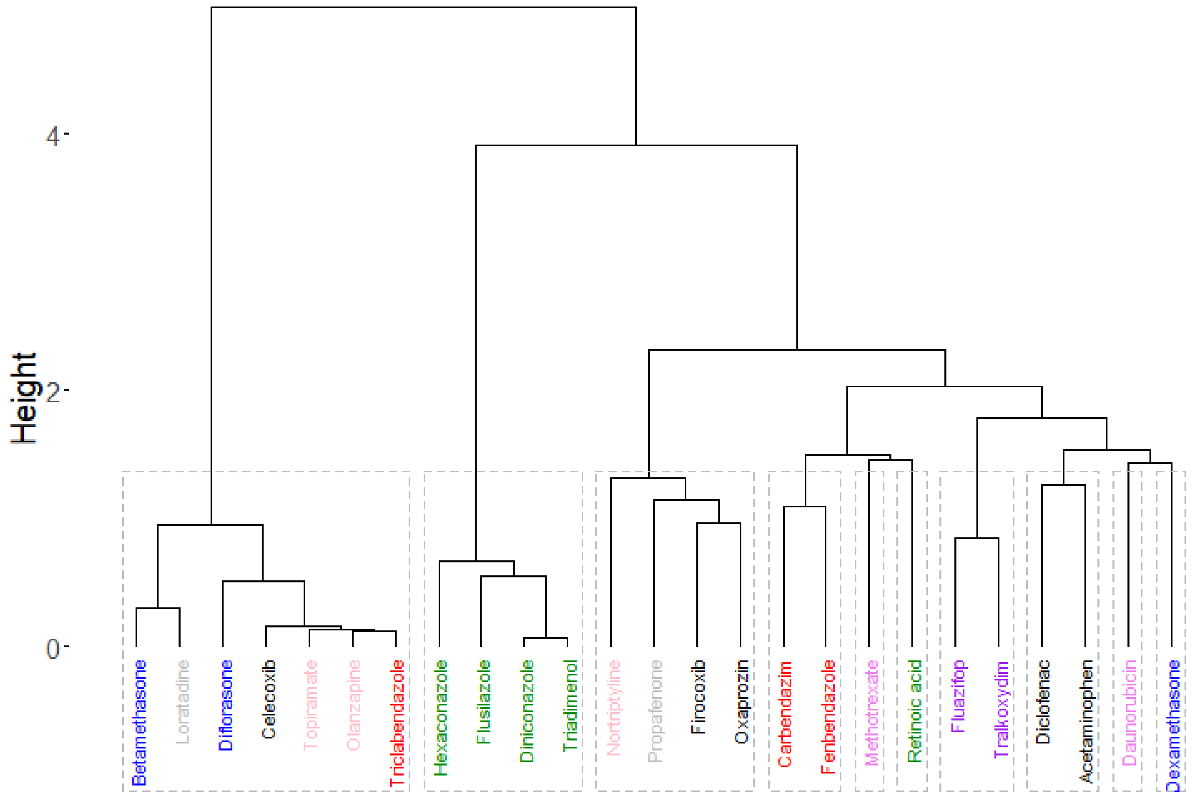

Fig. S4. Dendrogram of the cluster analysis using chemical $SR_{Baseline}$ based on the chord distance and ward method. Chemical names are colored according to their MoA group. (black: COX inhibitors, blue: glucocorticoids, red: tubulin inhibitors, purple: ACCase inhibitors, violet: antimitotics, grey: heart rate modulators, green: retinoic signaling interfering compounds, pink: neuroactive)

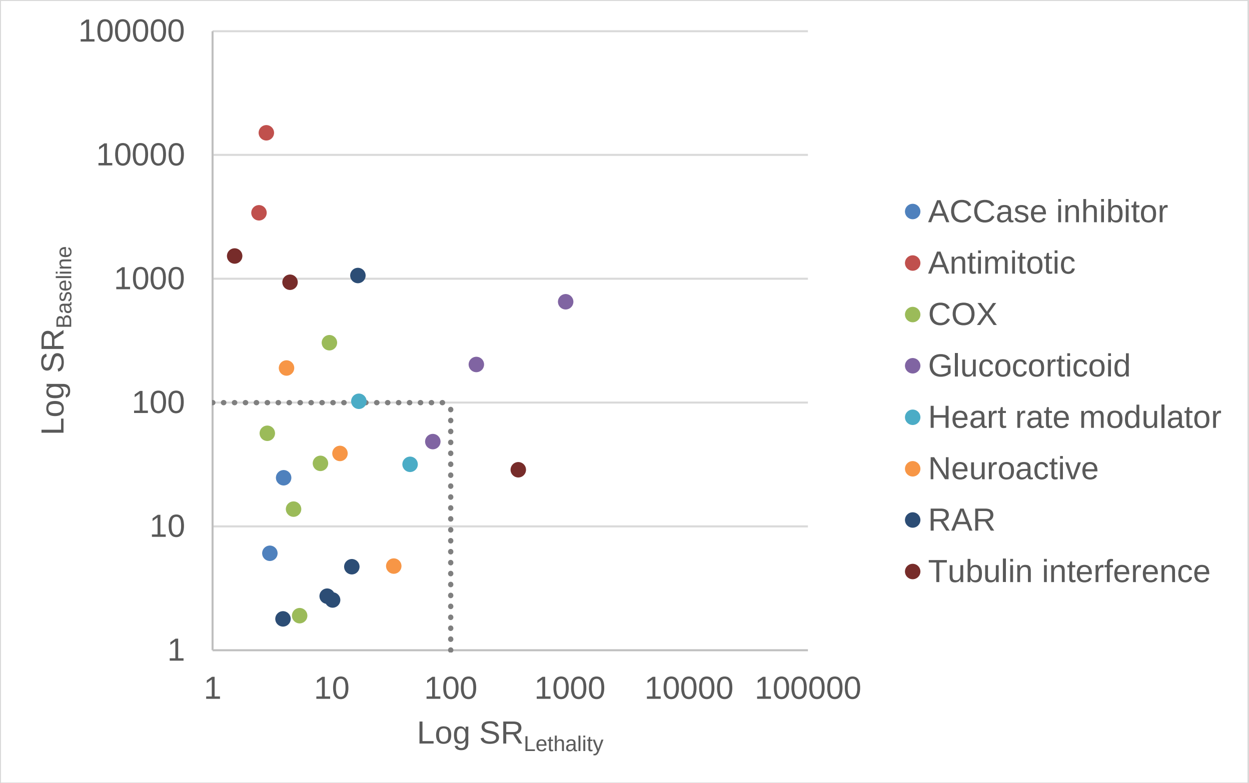

Fig. S5. Comparison of $SR_{Baseline}$ and $SR_{Lethality}$ of the most sensitive endpoint for each chemical tested in the study. Points are colored according to the chemical MoA group. RAR: chemicals interfering with the retinoic acid signaling. COX: cyclooxygenase inhibitors

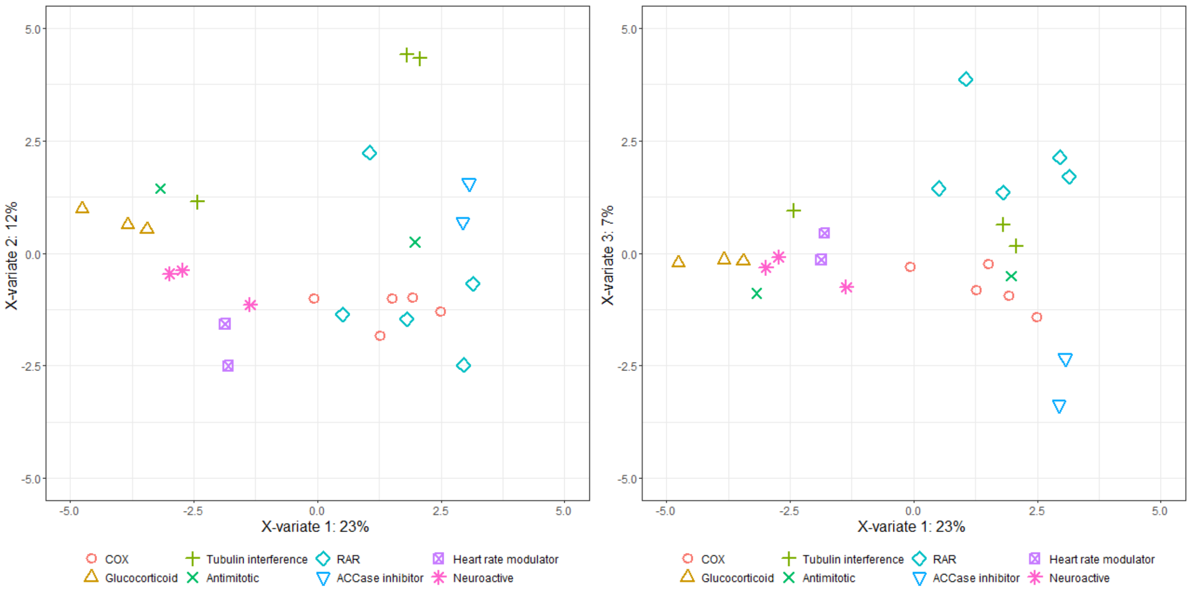

Fig. S6. PLS-DA score plots from the 30 endpoints analyzed with zebrafish using the $SR_{Baseline}$. Left figure shows score plot between component 1 and 2 and right figure between the component 1 and 3. Each dot represents a chemical and are grouped in 8 broad MoA classes. Accuracy of the model: 42.8%. RAR: chemicals interfering with the retinoic acid signaling. COX: cyclooxygenase inhibitors.

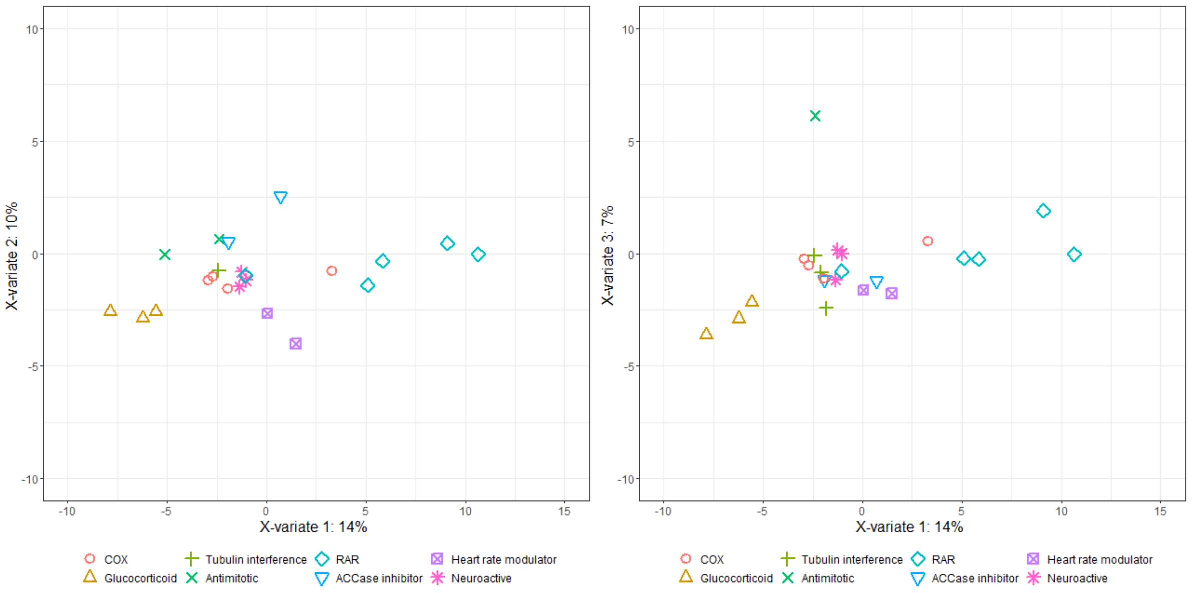

Fig. S7. PLS-DA score plot from the 30 endpoints analyzed with zebrafish using the $SR_{Lethality}$ combined with the 124 in vitro assays of ToxCast library using the $SR_{Cytotoxicity}$. Left figure shows score plot between component 1 and 2 and right figure between the component 1 and 3. Each dot represents a chemical and are grouped in 8 broad MoA classes. RAR: chemicals interfering with the retinoic acid signaling. COX: cyclooxygenase inhibitors

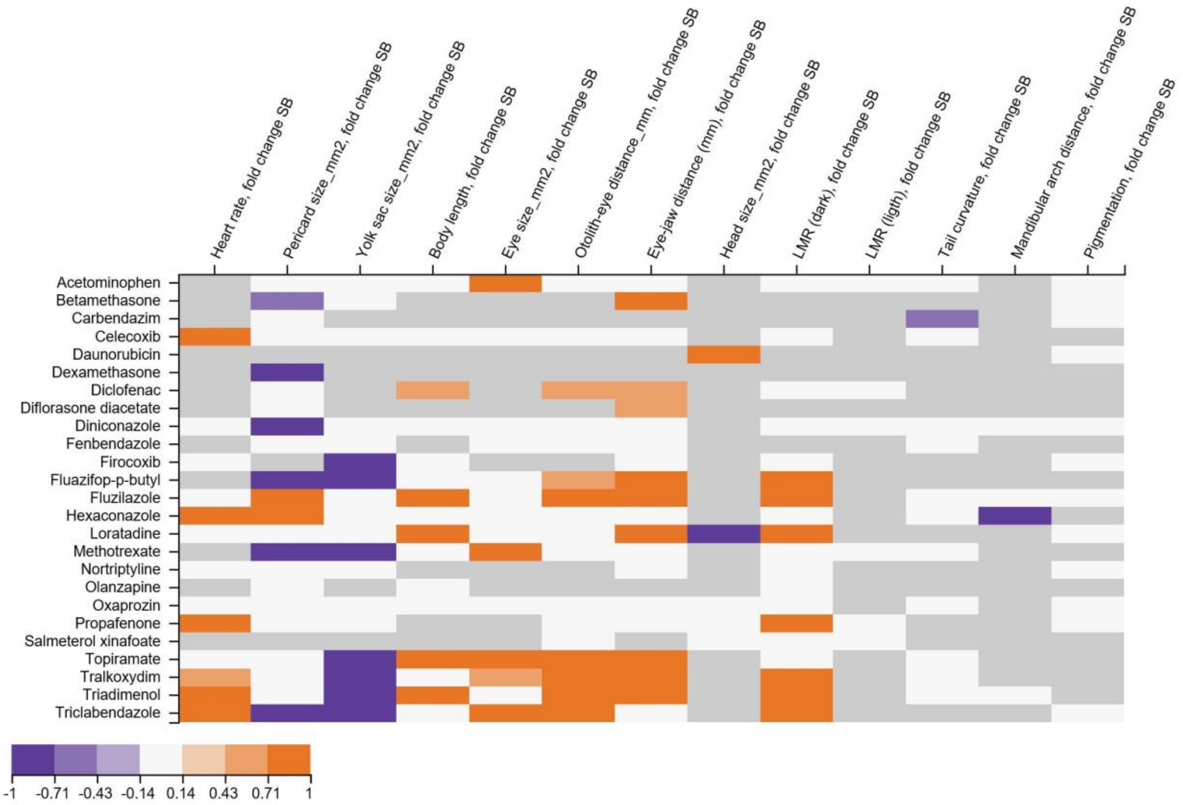

Fig. S8. Correlation between fold change in swim bladder size and other morphological and functional endpoints for each chemical (y-axis). Correlation is indicated as a color scale from purple (-1, negative correlation) to orange (1, positive correlation). Grey values depict no affected morphological and functional endpoints for each chemical.
